# Role of *TBX20* Truncating Variants in Dilated Cardiomyopathy and Left Ventricular Noncompaction

**DOI:** 10.1161/CIRCGEN.123.004404

**Published:** 2024-02-14

**Authors:** Almudena Amor-Salamanca, Alfredo Santana Rodríguez, Hazhee Rasoul, José F. Rodríguez-Palomares, Oana Moldovan, Thomas Morris Hey, María Gallego Delgado, David López Cuenca, Daniel de Castro Campos, María Teresa Basurte-Elorz, Rosa Macías-Ruiz, María Eugenia Fuentes Cañamero, Joseph Galvin, Raquel Bilbao Quesada, Luis de la Higuera Romero, Juan Pablo Trujillo-Quintero, Loida María García-Cruz, Ivonne Cárdenas-Reyes, Juan Jiménez-Jáimez, Soledad García-Hernández, María Valverde-Gómez, Iria Gómez-Díaz, Javier Limeres Freire, José M. García-Pinilla, Juan R. Gimeno-Blanes, Konstantinos Savattis, Pablo García-Pavía, Juan Pablo Ochoa

**Affiliations:** Cardiology Department, Health in Code SL, A Coruña, Spain (A.A.-S., L.d.l.H.R., I.C.-R., S.G.-H., M.V.-G., I.G.-D., J.P.O.).; Clinical Genetics Unit, Complejo Hospitalario Universitario Insular Materno Infantil, Las Palmas de Gran Canaria, Spain (A.S.R., L.M.G.-C.).; Research Institute of Biomedical and Health Sciences, University of Las Palmas de Gran Canaria, Spain (A.S.R., L.M.G.-C.).; Inherited Cardiovascular Diseases Unit, St. Bartholomew’s Hospital, Barts Health NHS Trust, London, United Kingdom (H.R., K.S.).; Cardiovascular Imaging Unit and Inherited Cardiac Diseases Unit, Cardiology Department, Vall d′Hebron University Hospital, Barcelona, Spain (J.F.R.-P., J.L.F.).; Vall d′Hebron Rsrch Unit, Barcelona, Spain (J.F.R.-P.).; Universitat Autònoma Barcelona, Spain (J.F.R.-P., J.P.T.-Q.).; Centro de Investigación Biomédica en Red en Enfermedades Cardiovasculares (CIBERCV), Instituto de Salud Carlos III, Madrid, Spain (J.F.R.-P., M.G.D., J.M.G.-P., J.R.G.-B., P.G.-P.).; European Reference Network for Rare and Low Prevalence Complex Diseases of the Heart, ERN GUARD-Heart, Amsterdam, The Netherlands (J.F.R.-P., J.L.F., J.R.G.-B., P.G.-P.).; Serviço de Genética Médica, Department de Pediatria, Hospital de Santa Maria, Centro Hospitalar Universitário Lisboa Norte, Portugal (O.M.).; Department of Cardiology, The Clinic of Inherited Cardiovascular Diseases, Odense University Hospital, Denmark (T.M.H.).; Cardiology Department, Hospital Universitario de Salamanca, Spain (M.G.D.).; Biomedical Research Institute of Salamanca, Gerencia Regional de Salud de Castilla y León, Spain (M.G.D.).; Department of Cardiology, Inherited Cardiac Diseases Unit, Hospital Clínico Universitario Virgen de la Arrixaca, Murcia, Spain (D.L.C., J.R.G.-B.).; Department of Cardiology, Heart Failure and Inherited Cardiac Diseases Unit, Hospital Universitario Puerta de Hierro, IDIPHISA, Madrid, Spain (D.d.C.C., P.G.-P., J.P.O.).; Department of Cardiology, Complejo Hospitalario de Navarra, Pamplona, Spain (M.T.B.-E.).; Cardiology Department, Hospital Universitario Virgen de las Nieves, Granada, Spain (R.M.-R., J.J.-J.).; Instituto de Investigación Biosanitaria Instituto de Investigación Biosanitaria de Granada (IBS-GRANADA), Spain (R.M.-R., J.J.-J.).; Cardiology Department, Hospital Universitario de Badajoz, Spain (M.E.F.C.).; Department of Cardiology, University College Dublin School of Medicine, Mater Misericordiae University Hospital, Ireland (J.G.).; Cardiology Department, Hospital Álvaro Cunqueiro, Vigo, Spain (R.B.Q.).; Center for Genomic Medicine, Parc Taulí Hospital Universitari, Sabadell, Spain (J.P.T.-Q.).; Institut d’Investigació i Innovació Parc Taulí, Sabadell, Spain (J.P.T.-Q.).; Inherited Cardiac Diseases Unit, Hospital Universitario San Cecilio, Granada, Spain (S.G.-H.).; Cardiology Department, Hospital Universitario 12 de Octubre, Madrid, Spain (M.V.-G.).; Department of Cardiology, Heart Failure and Inherited Cardiac Diseases Unit, Hospital Universitario Virgen de la Victoria, Instituto de Investigación Biomédica de Málaga (IBIMA), Málaga, Spain (J.M.G.-P.).; Department of Medicine and Dermatology, Universidad de Málaga, Spain (J.M.G.-P.).; Institute for Cardiovascular Science, University College London, United Kingdom (K.S.).; Biomedical Research Center, National Institute for Health and Care Research (NIHR) University College London Hospitals, United Kingdom (K.S.).; William Harvey Research Institute, Queen Mary University of London, United Kingdom (K.S.).; Universidad Francisco de Vitoria, Pozuelo de Alarcón, Spain (P.G.-P.).; Centro Nacional de Investigaciones Cardiovasculares, Madrid, Spain (P.G.-P., J.P.O.).

**Keywords:** cardiomyopathies, cardiomyopathy, dilated, heart defects, congenital, heart ventricles, high-throughput nucleotide sequencing, human genetics, transcription factors

## Abstract

**BACKGROUND:**

Less than 40% of patients with dilated cardiomyopathy (DCM) have a pathogenic/likely pathogenic genetic variant identified. *TBX20* has been linked to congenital heart defects; although an association with left ventricular noncompaction (LVNC) and DCM has been proposed, it is still considered a gene with limited evidence for these phenotypes. This study sought to investigate the association between the *TBX20* truncating variant (*TBX20tv*) and DCM/LVNC.

**METHODS:**

*TBX20* was sequenced by next-generation sequencing in 7463 unrelated probands with a diagnosis of DCM or LVNC, 22 773 probands of an internal comparison group (hypertrophic cardiomyopathy, channelopathies, or aortic diseases), and 124 098 external controls (individuals from the gnomAD database). Enrichment of *TBX20tv* in DCM/LVNC was calculated, cosegregation was determined in selected families, and clinical characteristics and outcomes were analyzed in carriers.

**RESULTS:**

*TBX20tv* was enriched in DCM/LVNC (24/7463; 0.32%) compared with internal (1/22 773; 0.004%) and external comparison groups (4/124 098; 0.003%), with odds ratios of 73.23 (95% CI, 9.90–541.45; *P*<0.0001) and 99.76 (95% CI, 34.60–287.62; *P*<0.0001), respectively. *TBX20tv* was cosegregated with DCM/LVNC phenotype in 21 families for a combined logarythm of the odds score of 4.53 (strong linkage). Among 57 individuals with *TBX20tv* (49.1% men; mean age, 35.9±20.8 years), 41 (71.9%) exhibited DCM/LVNC, of whom 14 (34.1%) had also congenital heart defects. After a median follow-up of 6.9 (95% CI, 25–75:3.6–14.5) years, 9.7% of patients with DCM/LVNC had end-stage heart failure events and 4.8% experienced malignant ventricular arrhythmias.

**CONCLUSIONS:**

*TBX20tv* is associated with DCM/LVNC; congenital heart defect is also present in around one-third of cases. *TBX20tv*-associated DCM/LVNC is characterized by a nonaggressive phenotype, with a low incidence of major cardiovascular events. *TBX20* should be considered a definitive gene for DCM and LVNC and routinely included in genetic testing panels for these phenotypes.

**See Editorial by Chang et al**

Nonischemic dilated cardiomyopathy (DCM) is characterized by enlargement of the left ventricle, usually accompanied by systolic dysfunction, not explained by abnormal loading conditions or significant coronary artery disease. Its prevalence is estimated to be closer to 1:250 individuals, and it is a leading cause of heart failure and heart transplantation worldwide.^1^ Left ventricular noncompaction (LVNC) is morphologically characterized by the presence of a thinned and compact myocardial layer and a thickened trabecular layer with excessive trabeculations and deep recesses.^2^ It is the third most common cardiomyopathy in the pediatric population after DCM and hypertrophic cardiomyopathy; it can also occur in the adult population with an estimated prevalence of 1:2000 individuals.^3^ LVNC has a broad morphological spectrum and can occur in isolation or association with other cardiomyopathies (mainly DCM and hypertrophic cardiomyopathy), early onset arrhythmias, or congenital heart defects (CHDs). Consequently, LVNC is a clinically heterogeneous disease, and affected patients can range from asymptomatic to severe cardiac dysfunction or sudden cardiac death.^2^

Since the introduction of next-generation sequencing in the late 20th century, many genes have been associated with the development of DCM/LVNC, with a constant effort to curate and update information on these genes. However, ≈60% to 70% of individuals with a clinical diagnosis of DCM/LVNC still do not have causal genetic variants identified, despite up to 40% of patients with genotype-negative DCM being reported as having familial disease.^4^

*TBX20* encodes the T-box transcription factor 20 protein, which is a member of the T-box superfamily that is highly expressed in embryonic heart tissues.^5,6^ Genetic variants in *TBX20* have been linked with CHD, and a potential association between variants in *TBX20* and the development of DCM in the absence of CHD has been proposed^7^; however, this association has not yet been definitively established. On the same hand, LVNC associated with *TBX20* variants has been described only in short reports.^8^ *TBX20* is currently considered a gene with limited evidence for DCM and LVNC.^9,10^

The objective of the present study was to explore the association of the *TBX20* truncating variant (*TBX20tv*) with DCM and LVNC phenotypes and to evaluate the characteristics and clinical outcomes of individuals with *TBX20tv*-associated DCM/LVNC.

## METHODS

Full methods of this research are available in Supplemental Methods.

The study conforms with the principles of the Declaration of Helsinki, and the study protocol was approved by the Independent Review Board of A Coruña-Ferrol (registry code 2022/435). Participants provided written informed consent. The authors from each center guaranteed the integrity of the data from their institution and received local approval for anonymized patient data collection and analysis.

All the data that support the findings of this study are available from the corresponding author upon reasonable request.

## RESULTS

### Enrichment of *TBX20tv* in DCM/LVNC

**Figure 1.**
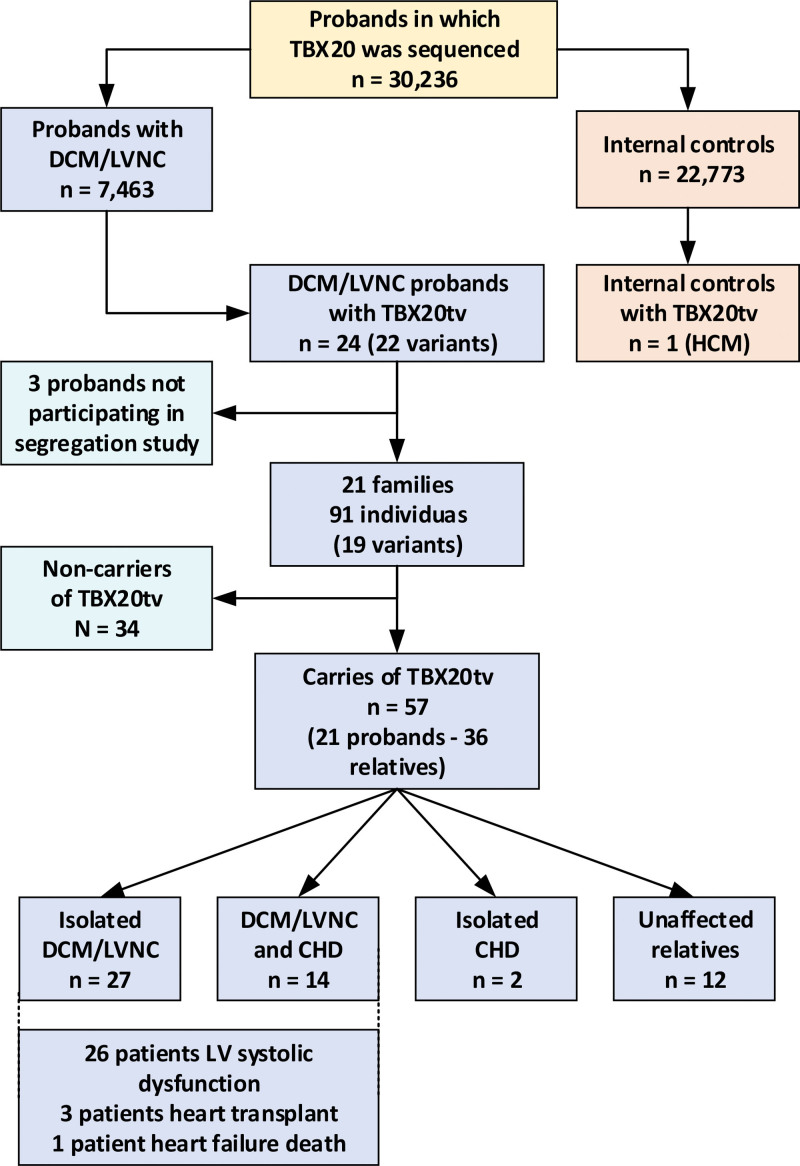
**Flowchart of the study.** Flowchart of the study after evaluating 30 236 probands in whom *TBX20* was sequenced. CHD indicates congenital heart defect; DCM, dilated cardiomyopathy; HCM, hypertrophic cardiomyopathy; LV, left ventricular; and LVNC, left ventricular noncompaction.

The flowchart of the study can be observed in Figure 1. We detected 22 different *TBX20tv* in 24 of 7463 DCM/LVNC probands. *TBX20tv* was significantly enriched in DCM/LVNC probands (24/7463; 0.32%) compared with internal (1/22 773; 0.004%) and external comparison groups (4/124 098; 0.003%), with an odds ratio of 73.23 (95% CI, 9.90–541.45; *P*<0.0001) and 99.76 (95% CI, 34.60–287.62; *P*<0.0001), respectively. The enrichment of *TBX20tv* was also demonstrated after performing a subgroup analysis, including DCM (isolated DCM and DCM with hypertrabeculation) and isolated LVNC, respectively (Table S1). Of these subgroups, the highest probability was obtained for the phenotype of DCM with hypertrabeculation, in which *TBX20tv* was detected in 5.26% of the probands with this phenotype (12/228 probands).

Of note, the only patient with a *TBX20tv* in the internal comparison group was a 65-year-old patient with hypertrophic cardiomyopathy with no left ventricular dilatation and normal ejection fraction (EF) but with a history of atrial fibrillation and nonsustained ventricular tachycardia; cardiac magnetic resonance imaging showed that he was positive for LGE. No other disease-causing variants except for the *TBX20tv* variant p.Arg420* were identified in the genetic study. This variant has been previously reported in the literature, identified in a family with a history of atrial septal defects (ASDs) but considered a VUS because it had been found to be a nonsegregating variant (one of the affected members with ASD was a noncarrier of the variant).^11^

A separate analysis to determine enrichment was performed for nontruncating variants in *TBX20*. We identified 31 probands with rare nontruncating variants in *TBX20* out of 7463 (0.41%) probands with DCL/LVNC, which, therefore, were not found to be significantly enriched compared with internal (95/22 694; 0.42%) and external (gnomAD) comparison groups (357/116 510; 0.031%), with an odds ratio of 0.99 (95% CI, 0.64–1.51; *P*=nonsignificant) and 1.36 (95% CI, 0.91–1.96; *P*=nonsignificant).

### Segregation Study, Penetrance, and Main Clinical Characteristics of *TBX20tv* Carriers

**Figure 2.**
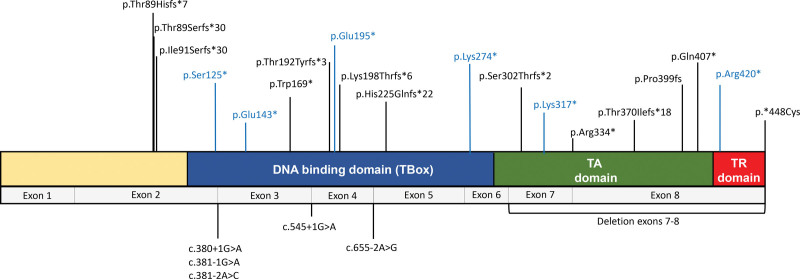
**TBX20 protein and localization of the variants detected in the study and reported in the literature.** Scheme of the TBX20 protein, with the relevant domains and coding exons, and the variants detected in the study (black) and described previously in the literature (blue). Truncating variants (nonsense and frameshift) are represented at the top of the figure, while splicing variants and CNVs are represented at the bottom. TA indicates transactivation; and TR, transrepression.

A total of 21 probands from the 24 individuals with DCM/LVNC and *TBX20tv* identified accepted to participate in the segregation and clinical evaluation studies; 9 of the probands (42.9%) had also a CHD in addition to DCM/LVNC. Patients were evaluated at 15 hospitals from 5 European countries: Spain (15 probands), United Kingdom (3 probands), Ireland (1 proband), Denmark (1 proband), and Portugal (1 proband). Of the 19 different loss-of-function variants identified, 9 were frameshift (47.37%), 3 nonsense (15.79%), and 5 affected splicing (26.32%); a stop-loss variant (5.26%) and a complete deletion of 2 exons (5.26%) were identified as well (Figure 2; Table S2).

In addition to the 21 probands of the families, clinical and genetic evaluations were undertaken in 70 relatives (range, 1–21 relatives per family). Thirty-six relatives (51.4%) were found to be carriers of *TBX20tv*, and a DCM/LVNC phenotype was found in 20 (58.8%), of whom 15 (75.0%) had an isolated DCM/LVNC and 5 (25.0%) had also a concomitant CHD. Twelve relative carriers of a *TBX20tv* (33.3%) were unaffected, and 2 (5.6%) had an isolated CHD (bicuspid aortic valve in both cases). The phenotype of the 2 carriers was unknown. None of the 34 relatives noncarriers of the *TBX20tv* present in the family had a diagnosis of DCM/LVNC or CHD.

Although most of the families were small, segregation analysis provided a combined logarythm of the odds score of 4.53, which is strongly indicative of the linkage between *TBX20tv* and DCM/LVNC.^12^ The most frequently identified *TBX20tv* was p.His225Glnfs*22, which had been detected in 15 patients from 2 large families coming from the same isolated geographic region in Spain. The identified *TBX20tv* was clearly cosegregated with DCM/LVNC in these families, with an independent logarythm of the odds score of 2.11. Pedigrees of all the families, logarythm of the odds scores, and the clinical characteristics of probands and affected relatives can be found in Table S4 and Figure S1, respectively.

**Figure 3.**
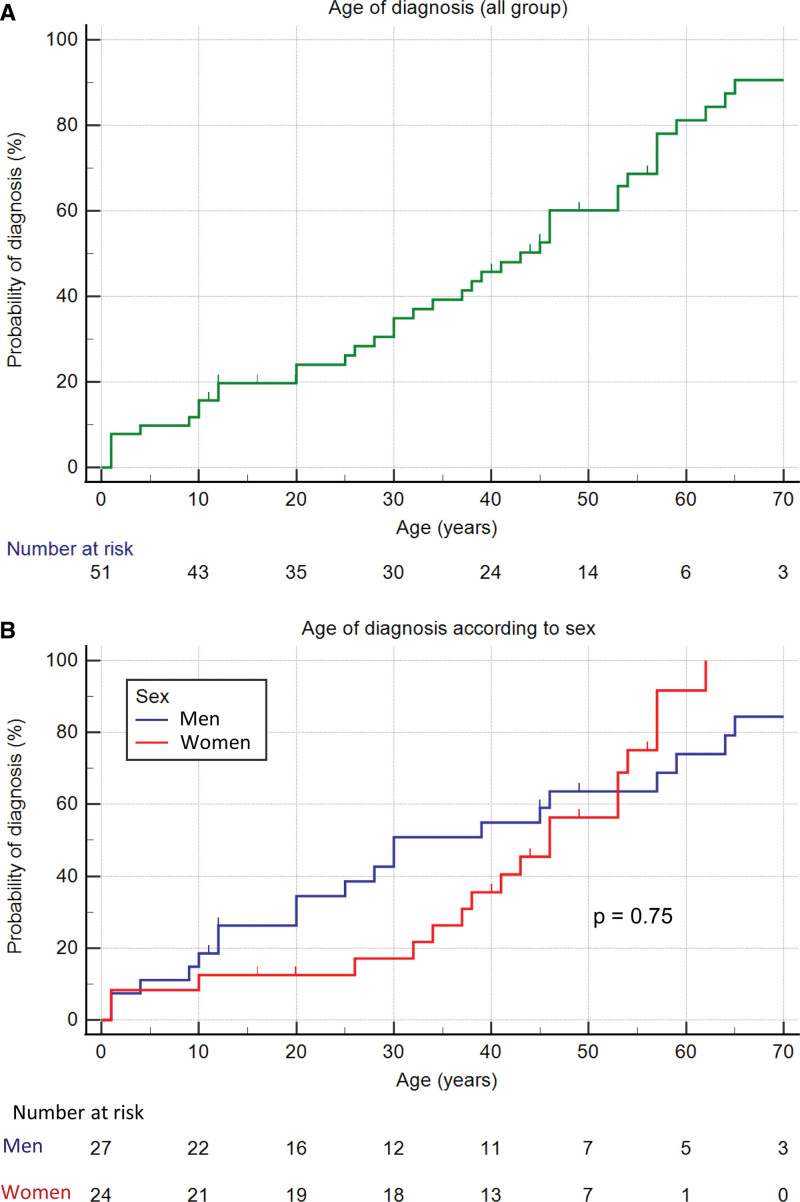
**Penetrance (age of diagnosis) of *TBX20tv*.** Penetrance of *TBX20tv* in the whole cohort (**A**) and in men and women (**B**). It can be observed how diagnosis begins early in life and is maintained constant through the years, with patients being diagnosed even after the age of 60 years.

Analysis of disease penetrance in all 57 individuals including probands and relatives harboring *TBX20tv* revealed an estimated median age of diagnosis in men of 30.0 (95% CI, 20.0–59.0) years and 46.0 (95% CI, 30.0–61.0) years in women (*P*=0.80; Figure 3). Interestingly, we also did not find differences in the age of onset of the disease between probands and relatives (Figure S2). On the other hand, the median age at diagnosis of patients with CHD in isolation or in combination with DCM/LVNC was 25.0 (95% CI, 9.0–45.0) years, while it was 43.0 (95% CI, 32.0–54.0) years in those with DCM/LVNC without CHD (*P*=0.08). Similarly, patients with LVNC were diagnosed earlier than those with isolated DCM (30.0 [95% CI, 12.0–41.0] versus 57.0 [95% CI, 10.0–73.0] years, respectively; *P*=0.054; Figure S2). Valvular involvement was the most common CHD (5 patients had bicuspid aortic valve and 5 had mitral valve defects), followed by heart septal defects (3 atrial and 3 ventricular), coronary anomalies (2), and aortic coarctation (2). Three patients had complex CHD: one patient had a ventricular septal defect, double outlet right ventricle, subaortic stenosis, and a single coronary artery; the second and third patients had both coarctation of the aorta and bicuspid aortic valve (one of them also with aortic dilatation).

Clinical characteristics and outcomes of patients with DCM/LVNC harboring *TBX20tv* demographic, clinical, echocardiographic, and electrocardiographic characteristics of the 41 patients with DCM/LVNC with *TBX20tv* are shown in the Table.

**Table.**
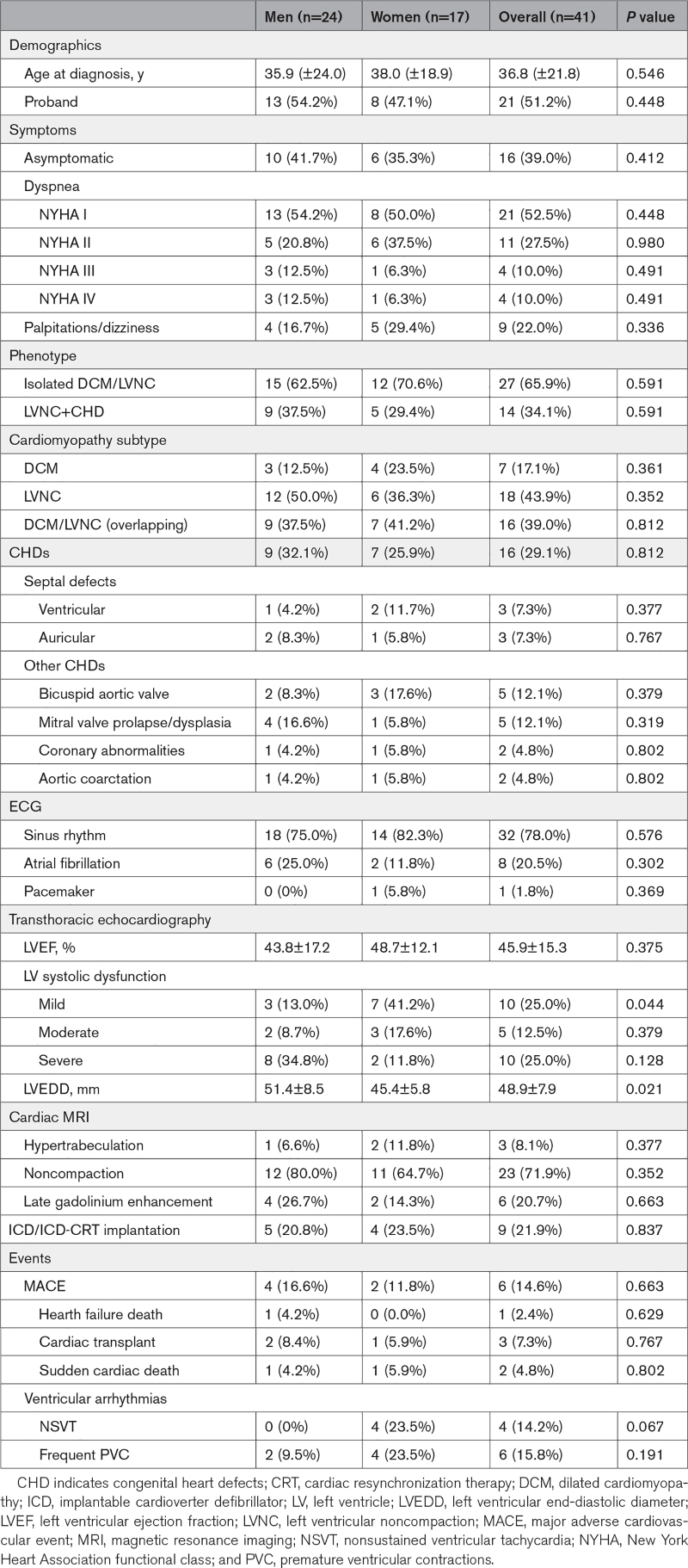
Characteristics of Patients With DCM/LVNC Carrying Truncating Variants in *TBX20*

**Figure 4.**
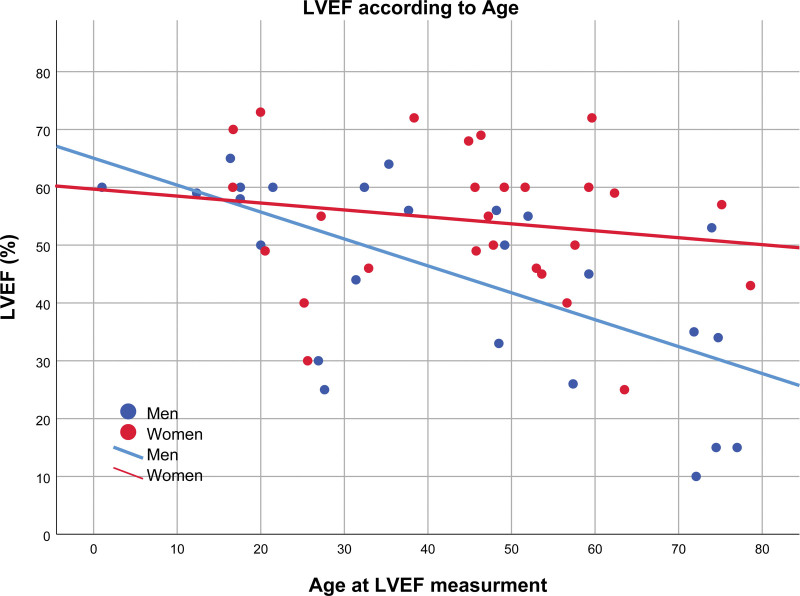
**Left ventricular ejection fraction (LVEF) according to the age of measurement.** Scatter plot representing the LVEF against the age in carriers of *TBX20tv* when the measurement was done. It can be observed how LVEF decreases over the years, being this phenomenon more pronounced in males.

The mean age at diagnosis was 36.8±21.8 years. The most frequent symptom was the presence of dyspnea (47.5%), with 20.0% of carriers being in NYHA classes III and IV. Palpitations or dizziness were present in 22.0% of the patients, and 39.0% were asymptomatic. Ventricular dysfunction (left ventricular EF, <55%) was identified in 62.5% of the carriers and was moderate to severe in 37.5% of the cases. There was no significant difference between the clinical presentations between men and women, except for higher left ventricular end-diastolic diameter in men (51.4±8.5 mm) than in women (45.4±5.8 mm; *P*=0.021). The relation between left ventricular EF and the age of the carriers of *TBX20tv* at which the measurement was performed can be observed in Figure 4. Left ventricular EF is decreasing at older ages, with this decrease being much more marked in men compared with women.

Events in carriers of *TBX20tv* with DCM/LVNC during a median follow-up of 6.9 (95% CI, 25–75:3.6–14.5) years were infrequent despite being severe in some cases. Survival curves free of major adverse cardiovascular events can be observed in Figure S3. Six patients (14.6%) experienced a major adverse cardiovascular event: 2 (4.8%) had major ventricular arrhythmia and 4 (9.7%) had end-stage heart failure. Three carriers (7.3%) required cardiac transplantation; they had in common the presence of CHD and a diagnosis at an early age. The first case was a boy diagnosed at 10 years of age with a bicuspid aortic valve and LVNC with a restrictive filling pattern. The second case was a male patient diagnosed with sinus venosus ASD at the age of 10 years. At 22 years, he began presenting symptoms of heart failure, progressing to ventricular dysfunction, a restrictive filling pattern, and pulmonary hypertension. He received a heart transplant at the age of 48 and died of postsurgical complications 1 week later. Finally, the third case was a 10-year-old girl initially diagnosed with hypertrophic cardiomyopathy with severe ventricular dysfunction and ASD. She had a complex genotype: de novo *TBX20tv* p.Lys198Thrfs*6 and 2 biallelic LP/P variants in *MYBPC3* inherited from each progenitor. The patient evolved unfavorably and required a heart transplant at the age of 24. The remaining carrier with end-stage heart failure died due to refractory heart failure at age 73, after surgery for an infrarenal aortic aneurysm with progressive worsening of his clinical situation, developing severe pulmonary hypertension. He had been diagnosed at 59 years of age with DCM/LVNC, being anticoagulated due to an apical thrombus (it was the only carrier with this complication in all the cohort).

The 2 patients with major ventricular arrhythmia events debuted with a recovered sudden cardiac death at 57 and 32 years of age, respectively; at the time of the event, only the male had systolic dysfunction (EF, 43%), but both evolved during follow-up with ventricular dilatation and dysfunction. Fibrosis on cardiac magnetic resonance imaging was not observed in either of the 2 cases.

The presence of other ventricular arrhythmias was infrequent in the cohort, with only 4 carriers (14.2%) presenting with nonsustained ventricular tachycardia and 6 (15.8%) with frequent ventricular extrasystoles. Nine carriers (21.9%) required the implantation of a defibrillator (7 in primary prevention) and 1 (1.8%) a conventional pacemaker. The prevalence of atrial fibrillation was 20.5% (8 patients), being higher in men (6 patients; 25.0%) than in women (2 patients; 11.8%) but not reaching statistical significance (*P*=0.30).

Fourteen carriers (34.1%) had isolated LVNC/hypertrabeculation (without CHD or dysfunction/dilatation); these patients had neither complications nor severe arrhythmias during follow-up (only 3 patients had a high burden of ventricular extrasystoles).

## DISCUSSION

In this multicenter study, we demonstrate a clear association between *TBX20tv* and DCM/LVNC. We documented a significant excess of loss-of-function variants in this gene among patients with DCM/LVNC compared with internal and external comparison groups and confirmed segregation of *TBX20tv* with DCM/LVNC in several families for a combined logarythm of the odds score of >3, which is highly indicative of significant linkage. Furthermore, we performed a deep characterization of clinical features and outcomes of *TBX20tv*-associated DCM/LVNC to realize that it follows a nonaggressive course, with a low incidence of ventricular arrhythmias, being the prognosis defined by progression to severe cardiac dysfunction and heart failure symptoms.

Genetic variants in *TBX20* had been proposed to cause DCM and LVNC, but most of the evidence for this association was based on functional studies and animal models.^13,14^ Clinical evidence was scarce, with disease-causing variants reported in small families and with variable segregation data.^15^ For these reasons, *TBX20* was considered a gene with limited evidence of association with DCM by the ClinGen curation consortium,^9,16^ and the association with LVNC has also not been considered definitive.^2^ A previous study about the genetic architecture of LVNC found significant enrichment of *TBX20tv* among patients with this phenotype although the number of patients was small and the authors did not consider the evidence to be sufficient to include this gene in the main section of the article (the analysis is given in the Supplemental Material).^17^

The first report that *TBX20* with cardiomyopathy in humans came from the work by Kirk et al.^18^ They described 2 variants in *TBX20*, p.Ile152Met and p.Glu195*, in 2 patients with both CHD and DCM. The nonsense truncating p.Glu195* was cosegregated into 6 members of a family with a mixed and variable phenotype that included ASD, mitral valve disease, and cardiomyopathy. Since then, several missense and few truncating variants have been identified in cohorts of patients with cardiomyopathy or isolated case reports, but cosegregation (with variable penetrance) was suggested for only a few of these variants.^7,19,20^

Our study provides solid evidence confirming that loss-of-function variants in *TBX20* are clearly associated with disease; according to our data, *TBX20tv* could explain at least 0.26% of cases with a diagnosis of DCM (isolated or with hypertrabeculation) and 0.75% of isolated LVNC cases. Our results also confirm that *TBX20* is a gene intolerant to haploinsufficiency, as it has been recently proposed by ClinGen^10^; this is relevant because new refinements of the American College of Medical Genetics criteria for specific conditions, such as DCM, are being proposed.^21^ In this sense, detecting a *TBX20tv* in a patient with DCM or LVNC should activate PVS1_Strong criteria and be considered likely pathogenic if it is also absent from controls and used predictively.

We did not find any association between nontruncating variants in *TBX20* and a DCM/LVNC phenotype. This does not necessarily mean that some rare nontruncating variants are not pathogenic; in fact, cosegregation has been demonstrated for a few variants but mainly with a CHD phenotype. As in other cardiomyopathy genes, these findings indicate that nontruncating variants have different effects—from gain to loss of function, including a neutral effect—and need to be evaluated separately and independently due to the absence of a class effect, as seems to happen with truncating variants. We cannot rule out the possibility that a few nontruncating variants are associated predominantly with DCM/LVNC, but the probability of explaining the phenotype when one of these variants is detected by genetic testing is lower than for truncating ones. From a practical point of view, the American College of Medical Genetics criteria PP2 (missense variant in a gene with a low rate of benign missense variants and common pathogenic missense variants) should not be applied to the *TBX20* gene.

It is also important to note that not all *TBX20tv* variants are necessarily equal in terms of their ultimate biological consequences. Although haploinsufficiency is probably the main final mechanism in most of these variants, there could be special cases in which the final products are viable proteins that can be associated with a different effect. For example, the stop-loss variant*448Cys only adds 3 new amino acids before a new stop codon, and nonsense variants in the last exon could escape the nonsense-mediated decay process, producing a slightly truncated protein (a fact that is not expected for nonsense variants in internal exons). On the same hand, whether any of the splicing variants can be associated with exon skipping and viable products is not known. One of the limitations of our work is the lack of functional studies exploring these possibilities, which could explain part of the variability observed in the phenotypes.

Regarding *TBX20tv*-associated clinical phenotype, an analysis of clinical and enrichment data according to the subphenotype suggests that *TBX20tv*-associated disease begins as noncompaction cardiomyopathy (which may be associated with CHD), progressing to a decrease in EF and dilatation, with several patients reaching a diagnosis of DCM. Almost half of the patients meet the criteria for DCM at the time of diagnosis although hypertrabeculation is also present in most cases.

TBX20 protein presents strong transcriptional activation and repression domains, and it interacts physically, functionally, and genetically with other cardiac developmental transcription factors, including NKX2-5, GATA4, GATA5, GATA6, and TBX5, which are associated with CHD and have also been linked to DCM/LVNC in recent years.^22–26^ Different growth factors involved in cardiac chamber development, ventricular trabeculation, and valvulogenesis upregulate and downregulate *TBX20* expression. Furthermore, functional studies in animal models have demonstrated that *TBX20* itself is important for cardiovascular development and function,^13,27^ which can partly explain why carriers of truncating variants in the gene initially present with an LVNC phenotype. On the same hand, it is, therefore, not surprising that variants in *TBX20* have been associated with CHD, with several functional studies supporting its relevance.^5,14^ Most of the variants associated with CHD are missense variants reported to cause septal defects.^28^ Valvular involvement has also been proposed in some papers in combination with septal defects.^29^ In our study, several patients with *TBX20tv* exhibited congenital septal defects, but valvular abnormalities in both the mitral valve and the aortic valve were more frequent than septal defects and occurred also in patients who did not have septal defects. Accordingly, our results suggest that the clinical phenotype of *TBX20tv* is heterogeneous and includes DCM/LVNC, valvular involvement, and bicuspid aortic valve, septal defects, and more complex CHD defects. Patients with LVNC and CHD were diagnosed earlier than those with isolated DCM, and many of them developed ventricular dilatation and dysfunction over time. In any case, it is important to remark that 2/3 (65.6%) of the patients in our cohort had DCM/LVNC with no evidence of any CHD.

Patients with *TBX20tv*-associated DCM/LVNC exhibit a benign clinical course, especially in terms of the arrhythmic risk, with <5% incidence of major ventricular arrhythmia. *TBX20tv*-associated DCM/LVNC was characterized by progressive left ventricular dysfunction and dilatation, with a prognosis characterized by progression to end-stage heart failure, observed in 9.7% of patients. These incidences of major adverse cardiovascular events are similar to or even lower than the incidence found in patients with negative genetic testing from large cohorts of DCM with similar follow-up times.^4^ It is worth noting that patients with isolated LVNC (without CHD or systolic dysfunction/dilatation) had no severe arrhythmias or cardiovascular events during follow-up.

Interestingly, although some other transcript factors, such as NKX2-5, have been associated with cardiomyopathy and septal defects and have also been linked with cardiac conduction disorders, we did not find an association of *TBX20tv* and cardiac conduction disorders, and only one of the patients of the cohort required pacemaker implantation due to sinus node dysfunction.

### Study Limitations

Limitations of the study include its observational nature and retrospective design. Our study cannot provide precise data on the prevalence of CHD associated with *TBX20tv* because all the probands in our study had a diagnosis of DCM/LVNC, and data on the prevalence of CHD among individuals with *TBX20tv* derive mainly from the evaluation of relatives.

Finally, this study was designed to prove the association of loss-of-function *TBX20* variants with DCM/LVNC, and our results do not necessarily reflect what happens with other variants such as nonsynonymous variants that can be associated with the gain of function.

### Conclusions

*TBX20tv* is associated with DCM/LVNC. The clinical course of the disease is not particularly aggressive, with a low number of patients evolving to end-stage heart failure or suffering major ventricular arrhythmia. Our results should lead to the incorporation of *TBX20* into the list of accepted DCM/LVNC-causing genes in humans. Moreover, *TBX20* should be included among the genes examined in patients with DCM, particularly among gene-elusive patients with concomitant hypertrabeculation or CHD.

## ARTICLE INFORMATION

### Acknowledgments

The authors thank Radek Suchac and Alicia Pallas for their help with English editing. The authors would also like to thank Elena Veira Villanueva for her help in constructing the family trees and Xusto Fernández Fernández, MD, for handling the family data. The authors also thank Núria Casanovas Marba, MD, and Neus Baena Díez, PhD, for the evaluation of patients at Parc Taulí Hospital Universitari.

### Sources of Funding

This study was funded by the Instituto de Salud Carlos III (ISCIII) through the projects PI17/01941 and PI20/01379 (cofunded by the European Regional Development Fund/European Social Fund: A Way to Make Europe/Investing in Your Future). The Centro Nacional de Investigaciones Cardiovasculares (CNIC) was supported by the ISCIII, Ministerio de Ciencia, Innovación y Universidades (MCIN), the Pro-CNIC Foundation, and the Severo Ochoa Centers of Excellence program (CEX2020-001041-S). The Hospital Universitario Puerta de Hierro, Virgen de la Arrixaca, and the Vall Hebron Hospital are members of the European Reference Network for Rare and Low Prevalence Complex Diseases of the Heart: ERN GUARD-Heart (http://guardheart.ern-net.eu).

### Disclosures

Drs Amor-Salamanca, de la Higuera Romero, Cárdenas-Reyes, García-Hernández, Valverde-Gómez, Gómez-Díaz, and Ochoa are employees of Health in Code SL. The other authors report no conflicts.

### Supplemental Material

Supplemental Methods

Tables S1–S4

Figures S1–S3

References 1,17,30–33

## Supplementary Material



## References

[1] Pinto YM Elliott PM Arbustini E Adler Y Anastasakis A Böhm M Duboc D Gimeno J de Groote P Imazio M. Proposal for a revised definition of dilated cardiomyopathy, hypokinetic non-dilated cardiomyopathy, and its implications for clinical practice: a position statement of the ESC working group on myocardial and pericardial diseases. Eur Heart J. 2016;37:1850–1858. doi: 10.1093/eurheartj/ehv727 26792875 10.1093/eurheartj/ehv727

[2] Rojanasopondist P Nesheiwat L Piombo S Porter GA Ren M Phoon CKL. Genetic basis of left ventricular noncompaction. Circ Genom Precis Med. 2022;15:e003517. doi: 10.1161/CIRCGEN.121.003517 35549379 10.1161/CIRCGEN.121.003517

[3] Towbin JA Jefferies JL. Cardiomyopathies due to left ventricular noncompaction, mitochondrial and storage diseases, and inborn errors of metabolism. Circ Res. 2017;121:838–854. doi: 10.1161/CIRCRESAHA.117.310987 28912186 10.1161/CIRCRESAHA.117.310987

[4] Escobar-Lopez L Ochoa JP Mirelis JG Espinosa MA Navarro M Gallego-Delgado M Barriales-Villa R Robles-Mezcua A Basurte-Elorz MT Gutiérrez García-Moreno L. Association of genetic variants with outcomes in patients with nonischemic dilated cardiomyopathy. J Am Coll Cardiol. 2021;78:1682–1699. doi: 10.1016/j.jacc.2021.08.039 34674813 10.1016/j.jacc.2021.08.039

[5] Sakabe NJ Aneas I Shen T Shokri L Park SY Bulyk ML Evans SM Nobrega MA. Dual transcriptional activator and repressor roles of TBX20 regulate adult cardiac structure and function. Hum Mol Genet. 2012;21:2194–2204. doi: 10.1093/hmg/dds034 22328084 10.1093/hmg/dds034 PMC3335310

[6] Carson CT Kinzler ER Parr BA. Tbx12, a novel T-box gene, is expressed during early stages of heart and retinal development. Mech Dev. 2000;96:137–140. doi: 10.1016/s0925-4773(00)00376-2 10940636 10.1016/s0925-4773(00)00376-2

[7] Zhao CM Sun B Song HM Wang J Xu WJ Jiang JF Qiu XB Yuan F Xu JH Yang YQ. TBX20 loss-of-function mutation associated with familial dilated cardiomyopathy. Clin Chem Lab Med. 2016;54:325–332. doi: 10.1515/cclm-2015-0328 26118961 10.1515/cclm-2015-0328

[8] Chang Y Wacker J Ingles J Macciocca I King I Semsarian C McGaughran J Weintraub RG Bagnall RD; Australian Genomics Cardiovascular Disorders Flagship. TBX20 loss-of-function variants in families with left ventricular non-compaction cardiomyopathy. J Med Genet. 2024;61:171–175. doi: 10.1136/jmg-2023-109455 37657916 10.1136/jmg-2023-109455

[9] Jordan E Peterson L Ai T Asatryan B Bronicki L Brown E Celeghin R Edwards M Fan J Ingles J. Evidence-based assessment of genes in dilated cardiomyopathy. Circulation. 2021;144:7–19. doi: 10.1161/CIRCULATIONAHA.120.053033 33947203 10.1161/CIRCULATIONAHA.120.053033 PMC8247549

[10] Rehm HL Berg JS Brooks LD Bustamante CD Evans JP Landrum MJ Ledbetter DH Maglott DR Martin CL Nussbaum RL; ClinGen. ClinGen--the clinical genome resource. N Engl J Med. 2015;372:2235–2242. doi: 10.1056/NEJMsr1406261 26014595 10.1056/NEJMsr1406261 PMC4474187

[11] Blue GM Kirk EP Giannoulatou E Dunwoodie SL Ho JWK Hilton DCK White SM Sholler GF Harvey RP Winlaw DS. Targeted next-generation sequencing identifies pathogenic variants in familial congenital heart disease. J Am Coll Cardiol. 2014;64:2498–2506. doi: 10.1016/j.jacc.2014.09.048 25500235 10.1016/j.jacc.2014.09.048

[12] Egeland T Pinto N Vigeland MD. A general approach to power calculation for relationship testing. Forensic Sci Int Genet. 2014;9:186–190. doi: 10.1016/j.fsigen.2013.05.001 23810238 10.1016/j.fsigen.2013.05.001

[13] Zhang W Chen H Wang Y Yong W Zhu W Liu Y Wagner GR Payne RM Field LJ Xin H. Tbx20 transcription factor is a downstream mediator for bone morphogenetic protein-10 in regulating cardiac ventricular wall development and function. J Biol Chem. 2011;286:36820–36829. doi: 10.1074/jbc.M111.279679 21890625 10.1074/jbc.M111.279679 PMC3196085

[14] Stennard FA Costa MW Lai D Biben C Furtado MB Solloway MJ McCulley DJ Leimena C Preis JI Dunwoodie SL. Murine T-box transcription factor Tbx20 acts as a repressor during heart development, and is essential for adult heart integrity, function and adaptation. Development. 2005;132:2451–2462. doi: 10.1242/dev.01799 15843414 10.1242/dev.01799

[15] Tayal U Prasad S Cook SA. Genetics and genomics of dilated cardiomyopathy and systolic heart failure. Genome Med. 2017;9:20. doi: 10.1186/s13073-017-0410-8 28228157 10.1186/s13073-017-0410-8 PMC5322656

[16] Urtis M Di Toro A Osio R Giuliani L Serio A Grasso M Fergnani V Smirnova A Aliberti F Arbustini E. Genetics and clinics: together to diagnose cardiomyopathies. Eur Heart J Suppl. 2022;24:I9–I15. doi: 10.1093/eurheartjsupp/suac097 36380800 10.1093/eurheartjsupp/suac097 PMC9653158

[17] Mazzarotto F Hawley MH Beltrami M Beekman L de Marvao A McGurk KA Statton B Boschi B Girolami F Roberts AM. Systematic large-scale assessment of the genetic architecture of left ventricular noncompaction reveals diverse etiologies. Genet Med. 2021;23:856–864. doi: 10.1038/s41436-020-01049-x 33500567 10.1038/s41436-020-01049-x PMC8105165

[18] Kirk EP Sunde M Costa MW Rankin SA Wolstein O Castro ML Butler TL Hyun C Guo G Otway R. Mutations in cardiac T-box factor gene TBX20 are associated with diverse cardiac pathologies, including defects of septation and valvulogenesis and cardiomyopathy. Am J Hum Genet. 2007;81:280–291. doi: 10.1086/519530 17668378 10.1086/519530 PMC1950799

[19] Vasilescu C Ojala TH Brilhante V Ojanen S Hinterding HM Palin E Alastalo TP Koskenvuo J Hiippala A Jokinen E. Genetic basis of severe childhood-onset cardiomyopathies. J Am Coll Cardiol. 2018;72:2324–2338. doi: 10.1016/j.jacc.2018.08.2171 30384889 10.1016/j.jacc.2018.08.2171

[20] Zhou YM Dai XY Huang RT Xue S Xu YJ Qiu XB Yang YQ. A novel TBX20 loss-of-function mutation contributes to adult-onset dilated cardiomyopathy or congenital atrial septal defect. Mol Med Rep. 2016;14:3307–3314. doi: 10.3892/mmr.2016.5609 27510170 10.3892/mmr.2016.5609

[21] Morales A Kinnamon DD Jordan E Platt J Vatta M Dorschner MO Starkey CA Mead JO Ai T Burke W; DCM Precision Medicine study of the DCM Consortium. Variant interpretation for dilated cardiomyopathy: refinement of the American College of Medical Genetics and Genomics/ClinGen Guidelines for the DCM Precision Medicine Study. Circ Genom Precis Med. 2020;13:e002480. doi: 10.1161/CIRCGEN.119.002480 32160020 10.1161/CIRCGEN.119.002480 PMC8070981

[22] Zhang XL Dai N Tang K Chen YQ Chen W Wang J Zhao CM Yuan F Qiu XB Qu XK. GATA5 loss-of-function mutation in familial dilated cardiomyopathy. Int J Mol Med. 2015;35:763–770. doi: 10.3892/ijmm.2014.2050 25543888 10.3892/ijmm.2014.2050

[23] Yuan F Qiu XB Li RG Qu XK Wang J Xu YJ Liu X Fang WY Yang YQ Liao DN. A novel NKX2-5 loss-of-function mutation predisposes to familial dilated cardiomyopathy and arrhythmias. Int J Mol Med. 2015;35:478–486. doi: 10.3892/ijmm.2014.2029 25503402 10.3892/ijmm.2014.2029

[24] Xu L Zhao L Yuan F Jiang WF Liu H Li RG Xu YJ Zhang M Fang WY Qu X-K. GATA6 loss-of-function mutations contribute to familial dilated cardiomyopathy. Int J Mol Med. 2014;34:1315–1322. doi: 10.3892/ijmm.2014.1896 25119427 10.3892/ijmm.2014.1896

[25] Zhao L Xu JH Xu WJ Yu H Wang Q Zheng HZ Jiang WF Jiang JF Yang YQ. A novel GATA4 loss-of-function mutation responsible for familial dilated cardiomyopathy. Int J Mol Med. 2014;33:654–660. doi: 10.3892/ijmm.2013.1600 24366163 10.3892/ijmm.2013.1600

[26] Zhou W Zhao L Jiang JQ Jiang WF Yang YQ Qiu XB. A novel TBX5 loss-of-function mutation associated with sporadic dilated cardiomyopathy. Int J Mol Med. 2015;36:282–288. doi: 10.3892/ijmm.2015.2206 25963046 10.3892/ijmm.2015.2206

[27] Chen Y Xiao D Zhang L Cai CL Li BY Liu Y. The role of Tbx20 in cardiovascular development and function. Front Cell Dev Biol. 2021;9:638542. doi: 10.3389/fcell.2021.638542 33585493 10.3389/fcell.2021.638542 PMC7876368

[28] Liu C Shen A Li X Jiao W Zhang X Li Z. T-box transcription factor TBX20 mutations in Chinese patients with congenital heart disease. Eur J Med Genet. 2008;51:580–587. doi: 10.1016/j.ejmg.2008.09.001 18834961 10.1016/j.ejmg.2008.09.001

[29] Posch MG Gramlich M Sunde M Schmitt KR Lee SHY Richter S Kersten A Perrot A Panek AN Al Khatib IH. A gain-of-function TBX20 mutation causes congenital atrial septal defects, patent foramen ovale and cardiac valve defects. J Med Genet. 2010;47:230–235. doi: 10.1136/jmg.2009.069997 19762328 10.1136/jmg.2009.069997 PMC2981023

[30] Lek M Karczewski KJ Minikel EV Samocha KE Banks E Fennell T O’Donnell-Luria AH Ware JS Hill AJ Cummings BB; Exome Aggregation Consortium. Analysis of protein-coding genetic variation in 60,706 humans. Nature. 2016;536:285–291. doi: 10.1038/nature19057 27535533 10.1038/nature19057 PMC5018207

[31] Whiffin N Minikel E Walsh R O’Donnell-Luria AH Karczewski K Ing AY Barton PJR Funke B Cook SA Macarthur D. Using high-resolution variant frequencies to empower clinical genome interpretation. Genet Med. 2017;19:1151–1158. doi: 10.1038/gim.2017.26 28518168 10.1038/gim.2017.26 PMC5563454

[32] Arbelo E Protonotarios A Gimeno JR Arbustini E Barriales-Villa R Basso C Bezzina CR Biagini E Blom NA de Boer RA; ESC Scientific Document Group. 2023 ESC guidelines for the management of cardiomyopathies. Eur Heart J. 2023;44:3503–3626. doi: 10.1093/eurheartj/ehad194 37622657 10.1093/eurheartj/ehad194

[33] Richards S Aziz N Bale S Bick D Das S Gastier-Foster J Grody WW Hegde M Lyon E Spector E; ACMG Laboratory Quality Assurance Committee. Standards and guidelines for the interpretation of sequence variants: a joint consensus recommendation of the American College of Medical Genetics and Genomics and the Association for Molecular Pathology. Genet Med. 2015;17:405–424. doi: 10.1038/gim.2015.30 25741868 10.1038/gim.2015.30 PMC4544753

