# Supplementary material for: Role of TBX20 Truncating Variants in Dilated Cardiomyopathy and Left Ventricular Noncompaction
Source: Circ Genom Precis Med. 2024 Feb 14;17(2):e004404. doi: 10.1161/CIRCGEN.123.004404 (PMC11019988; doi:10.1161/CIRCGEN.123.004404)

**Supplemental Material**

**The Role of TBX20-Truncating Variants in Dilated Cardiomyopathy and Left Ventricular Non-Compaction**

**Amor Salamanca et al.**

**Table of contents:**

Supplementary methods…………………………………………………………………..………………....2

Variant classification and rules applied for categorization…...…………………………........................7

List of genes included in the sequencing library………..............……………………………………….11

Table S1: Enrichment of TBX20tv in different subgroups…………….……….…………...……………16

Table S2: TBX20tv variants detected in the study….........................................................................17

Table S3: Other potentially relevant variants identified in the present study.....................................18

Table S4: Clinical characteristics of all the carriers……………………….……………….….…..…..…19

Figure S1: Pedigrees of TBX20tv carriers………………………………………………….....................24

Figure S2: Penetrance according to index status and phenotype.....................................................32

Figure S3: Survival free of MACE......................................................................................................33

**Supplementary Methods**

***Design and study population:*** This was a multicenter, retrospective longitudinal cohort study. The study conforms with the principles of the Declaration of Helsinki, and the study protocol was approved by the Independent Review Board of A Coruña-Ferrol (registry code 2022/435). Participants provided written informed consent. The authors from each center guaranteed the integrity of the data from their institution and received local approval for anonymized patient data collection and analysis.

From February 2014 to December 2022, the *TBX20* gene was sequenced by NGS in 30,236 consecutive unrelated probands with a diagnosis of different inherited cardiac conditions referred to our center for molecular genetic diagnosis. The phenotypes were established by each center prior to the genetic studies. Patients’ samples were referred mainly from centers from Spain, followed by centers from the United Kingdom, Denmark, United States, Germany, Ireland, Portugal and Argentina. A total of 7,463 probands (24.6%) had a diagnosis of DCM or LVNC, while 22,773 (75.3%) were considered internal controls with other phenotypes and no evidence of DCM/LVNC (mainly HCM, channelopathies and aortic diseases). The predominant ethnicity was European (>90% of the probands), with no differences between DCM/LVNC probands and controls. Additionally, individuals from gnomAD database version v2.1.1, Dec 2022 (“https://gnomad.broadinstitute.org/”), were used as external controls.^32^

*TBX20* rare variants were identified, and their frequencies compared between DCM/LVNC probands and control groups. The analysis was performed for truncating and non-truncating variants (see below for definitions). We applied a MAF threshold of 8x10^-5^ to consider a variant a candidate, as previously described in order to reassess gene pathogenicity in cardiomyopathies,^33^ and excluded variants with a MAF ≥1x10^-4^ in any sub-population defined in gnomAD to avoid that variants detected in cases could be enriched in a specific population (none of the *TBX20tv* identified in this study had a MAF >8x10^-5^).

Subsequently, we invited identified probands with *TBX20tv* and their relatives to participate in clinical and segregation studies. Clinical and genetic familial cascade screening was performed following written informed consent in those who agreed to participate. The clinical characteristics and outcomes in carriers of *TBX20tv* (including probands and relatives) were assessed. Baseline demographics, comorbidities, symptoms, 12-lead electrocardiogram (ECG), transthoracic echocardiogram (TTE), ambulatory Holter ECG recordings and cardiac magnetic resonance (CMR) scan data were collected from clinical records.

Clinical diagnosis of DCM was based on the finding of a left ventricular ejection fraction (LVEF) <50% not explained by abnormal loading conditions or coronary artery disease.^34^ Following the latest recommendations, a patient was considered to have DCM independently of the presence of associated LVNC/hypertrabeculation.^35^ LVNC was defined as the presence of significant hypertrabeculation in the left ventricle with a ratio between the non-compacted/compacted wall ≥2.3; the presence of non-compaction with a ratio <2.3 was considered hypertrabeculation without a definitive diagnosis of LVNC.^36^

Due to the overlap between the DCM and LVNC phenotypes and in the absence of a consensus classification, the following definitions were used in this paper:

- **DCM:** as described above, the patient meets DCM criteria, independently of the presence of LVNC/hypertrabeculation (includes isolated DCM and DCM with hypertrabeculation; see below).
- **Isolated DCM:** meets DCM criteria without evidence of hypertrabeculation.
- **DCM with hypertrabeculation:** meets DCM criteria and also shows significant hypertrabeculation (LVNC or hypertrabeculation).
- **Isolated LVNC/hypertrabeculation:** does not meet DCM criteria but shows morphologic criteria for LVNC or hypertrabeculation.
- **DCM/LVNC:** any patient that meets criteria for DCM and/or LVNC/hypertrabeculation (includes all the previous groups).

***Genetic studies, variant filtering and variant classification:*** Coding exons and intronic boundaries of 213 genes related to inherited cardiovascular diseases and sudden cardiac death (SCD) (Supplementary Methods) were captured using a custom probe library (SureSelect Target Enrichment Kit for Illumina paired-end multiplexed sequencing method; Agilent Technologies, Santa Clara, California, USA) and sequenced using a HiSeq 1500 or a NovaSeq 6000 platform (Illumina, San Diego, California, USA). The read depth (number of times that a base was sequenced by independent reads) of every nucleotide of genes related to the referring phenotype (including *TBX20*) was greater than 30x (mean coverage 250-400x). Exons that did not fulfill this standard were complementary sequenced using the Sanger method. Bioinformatics analysis was performed by means of a custom pipeline including software for variant calling, genotyping and annotation. All the variants identified were referred to the canonical transcript of *TBX20* (RefSeq NM_001077653.2 and NP_001071121.1; ENSEMBL ENSG00000164532).

A variant in *TBX20* was considered “truncating” if software tools predicted the appearance of a premature stop codon at the protein level (nonsense and frameshift variants) or if it affected the canonical splice acceptors or donors of introns of the gene (cDNA positions -2 and -1, and +1 and +2); CNVs in *TBX20* (gene/exon deletions) and stop-loss variants were also included if the evidence was supportive of pathogenicity. Nonsynonymous variants (missense) and small in-frame indels (insertions or deletions of nucleotides that do not shift the reading frame, resulting in the loss or addition of fewer than three amino acids in the final protein sequence) were considered “non-truncating” variants.

To establish the pathogenicity of identified variants, we developed a customized classification scheme based on the recommendations of the American College of Medical Genetics and Genomics (Supplementary Methods);^37^ the final classification of each variant was agreed by consensus between two cardiologists with experience in interpretation of genetic variants (AAS and JPO).

***Statistics:*** Continuous variables were expressed as mean ± SD, and comparison between groups was performed using the Student T test or the Mann-Whitney test, according to values distribution. Non-continuous variables were expressed as an integer number (percent of total) and compared using the chi-square test or Fisher’s exact test, as appropriate. A two-sided p value <0.05 was considered to indicate statistical significance. Analysis was performed using R version 3.4.3 (The R Foundation for Statistical Computing Platform).

***Segregation and LOD (Logarithms of Odds) score:*** We calculated two-point LOD score for 15 informative families (Figure S1) by using the PARAMLINK package for R software. The model was set with θ = 0, phenocopy rate = 0.005 and a penetrance value of 0.80. Only patients with a diagnosis of DCM/LVNC were considered affected (patients with isolated CHD and no evidence of cardiomyopathy were considered unaffected for the analysis to calculate specifically the linkage with DCM/LVNC). An indeterminate status was assigned to family members who were not clinically evaluated, as well as to males <45 years and females <50 years who did not meet clinical criteria for DCM/LVNC and could develop the disease afterwards.

***Survival analysis and disease penetrance:*** The cumulative probability of cardiovascular death in carriers of *TBX20tv* after a diagnosis of DCM/LVNC was estimated using the Kaplan-Meier method. Major cardiovascular events (MACE) were defined as the presence of malignant ventricular arrhythmias (MVA), including SCD or appropriate defibrillator shock, or end-stage heart failure events (ESHF), including heart failure death or heart transplantation. The beginning of the follow-up was established as the first clinical evaluation. Patients were censored at the time of their first endpoint event during follow-up or at their last evaluation. A cumulative probability plot was used to examine age at disease penetrance, defined as receiving a diagnosis of DCM, LVNC or CHD.

**Variant classification and rules applied for categorization**

Rare genetic variants detected in probands were centrally assessed for pathogenicity and subsequently classified as pathogenic (P), likely pathogenic (LP), variant of uncertain significance (VUS), likely benign (LB) or benign (B) by expert assessment using modified criteria of the American College of Medical Genetics and Genomics and the Association for Molecular Pathology (ACMG/AMP) guidelines following the recommendations of the ClinGen Guidelines for Variant Interpretation in Dilated Cardiomyopathy (Morales A. et al. Variant interpretation for dilated cardiomyopathy. *Circulation: Genomic and Precision Medicine*, 43–51).

The following ACMG/AMP modified rules were applied:

***Population frequency:***

**PM2:** Applied if the filtering allele frequency in the Genomes Aggregation Database (gnomAD) exomes dataset (version 2.1) was below 0.004%, and if the variant was absent from a cohort of disease-controls of Spanish origin (Health in Code database).

**BS1:** Applied if the filtering allele frequency in the gnomAD exomes dataset (version 2.1) was ≥0.05%.

**BA1:** Applied if the filtering allele frequency in the gnomAD exomes dataset (version 2.1) was >0.1%.

***General rules:***

**PVS1:** Truncating variants, i.e., frameshift, nonsense, splice donor and splice acceptor variants, initiation codon, single or multi-exon deletion in the following genes in which LOF is a proved mechanism of disease: *DSP, PKP2, LMNA, MYBPC3, FHL1, DMD*.

**PVS1_Strong:** Truncating variants, i.e., frameshift, nonsense, splice donor and splice acceptor variants, initiation codon, single or multi-exon deletion in the following genes in which LOF is a proved mechanism of disease: *DSG2, DSC2, FLNC, TNNT2, PLN, NKX2-5, PRDM16*. In *TTN*, the rule applied if the variant affected the A band and/or constitutive exons in the adult cardiac N2B isoform (more than 95% of exon usage -transcript incorporation- in human adult left ventricle).

**PM4/BP3:** A protein length change because of an in-frame deletion or insertion in a non-repeat region or within a region annotated by repeat masker.

**PS1:** Same amino acid change as a previously established pathogenic variant (multiple ClinVar submissions with no conflicting evidence).

**PM5:** Novel missense change at an amino acid residue where a different missense change has previously been established as pathogenic (multiple ClinVar submissions with no conflicting evidence).

**PM1:** Located in a mutational hot spot and/or critical and well-established functional domain (e.g., active site of an enzyme) without benign variation. This rule applied only for non-truncating rare variants (PM2 rule activated) present in genes that had regions/domains in which all missense variants in these domains identified to date have been shown to be pathogenic and specifically associated with DCM. The rules applied only for RBM20 (amino acid 630-640 and 910-920), and TNNT2 (residues 131-179). We considered that in the remaining genes, the information in relation to DCM was insufficient to apply this rule, and that the information based on other phenotypes (i.e. HCM) should not be extrapolated.

***Case-control analysis and probands with a consistent phenotype (only one of the following rules can be activated):***

**PS4:** Two scenarios are contemplated:

1. Variants enriched in case cohorts compared with population controls. The DCM/LVNC cohort used was the Health in Code, A Coruña, Spain, comprising up to 7,463 probands (enrichment was defined as a Fisher’s exact test *P* < 1.79 x 10^-6^, after multiple testing correction). PS4 was also applied for any additional variants enriched in the DCM cohort described in this study (present in ≥3 cases and *P* < 1.9 x 10^-4^ after Bonferroni multiple testing correction).
2. ≥15 probands with a consistent confirmed phenotype (DCM). Applies only for very rare variants (PM2 criteria met).

**PS4_Moderate:** Variant identified in ≥6 probands with consistent confirmed phenotypes (DCM). Only applicable if the variant is absent or rare in large population studies (PM2 criteria met).

**PS4_Supporting:** Variant identified in ≥2 probands with consistent confirmed phenotypes (DCM). Only applicable if the variant is absent or rare in large population studies (PM2 criteria met).

***Cosegregation rules (only one of the following rules can be activated):***

**PP1_Strong:** Cosegregation with disease in ≥7 segregations in affected family members in a gene definitively known to cause the disease (present in ClinGen curation for DCM). Only applicable if the variant is also absent or rare in large population studies (PM2 criteria met).

**PP1_Moderate:** Cosegregation with disease in ≥5 segregations in affected family members in a gene definitively known to cause the disease (present in ClinGen curation for DCM). Only applicable if the variant is also absent or rare in large population studies (PM2 criteria met).

**PP1:** Cosegregation with disease in ≥3 segregations in affected family members in a gene definitively known to cause the disease (present in ClinGen curation for DCM). Only applicable if the variant is also absent or rare in large population studies (PM2 criteria met).

***De novo rules:***

**PS2:** De novo (paternity confirmed) in a patient with the disease and no family history (FH) (no suspicion of cardiomyopathy through three generations, and parents have been thoroughly clinically evaluated without evidence suggestive of cardiomyopathy). Only applicable in the ABSENCE of any other possible disease-causing variants.

**PM6:** Confirmed de novo, but without confirmation of paternity or maternity. Both parents must have been tested and shown not to carry the variant, but clinical evaluation of parents is not required.

***Functional studies and predictors:***

**PS3:** Well-established *in vitro* or *in vivo* functional studies supportive of a damaging effect on the gene or gene product. Mammalian variant-specific knock-in models or cell model (or other in vitro assay) that produces a cellular phenotype that reliably predicts clinical DCM, or causality is demonstrated with appropriate controls (e.g., correction of the variant reverses the phenotype).

**PP3/BP4:** Multiple lines of computation evidence support or refute a deleterious effect.

For missense variants, CADD, DANN, FATHMM, Polyphen-2, and MutationTaster were used. The rule applied if 4/5 predictors yield the same result on the impact on the protein.

For intronic variants out of the splicing consensus site (+1, +2, -1, -2) the following predictors were used: SSF, MaxEnt, NNSplice, GeneSplicer and AdaBoost. The rule applied if 4/5 predictors yield the same result on the impact on splicing.

**List of 213 genes related to inherited cardiovascular diseases and sudden death included in the custom probe library.**

| Name | **Description** |
| --- | --- |
| *AARS2* | Alanine--tRNA ligase, mitochondrial |
| *ABCC9* | ATP-binding cassette, sub-family C (CFTR/MRP), member 9 |
| *ACAD9* | Acyl-CoA dehydrogenase family member 9, mitochondrial |
| *ACADM* | Medium-chain specific acyl-CoA dehydrogenase, mitochondrial |
| *ACADVL* | Very long-chain specific acyl-CoA dehydrogenase, mitochondrial |
| *ACTA1* | Actin, alfa 1, skeletal muscle |
| *ACTA2* | Actin, aortic smooth muscle |
| *ACTC1* | Actin, alpha cardiac muscle 1 |
| *ACTN2* | Alpha-actinin-2 |
| *ACVRL1* | Serine/threonine-protein kinase receptor R3 |
| *ADAMTSL4* | ADAMTS-like protein 4 |
| *AGK* | Acylglycerol kinase, mitochondrial |
| *AGL* | Glycogen debranching enzyme |
| *AGPAT2* | 1-acyl-sn-glycerol-3-phosphate acyltransferase beta |
| *AKAP9* | A-kinase anchor protein 9 |
| *ALMS1* | Alstrom syndrome protein 1 |
| *ANK2* | Ankyrin 2 |
| *ANK3* | Ankyrin-3 |
| *ANKRD1* | Ankyrin repeat domain-containing protein 1 |
| *APOA5* | Apolipoprotein A-V |
| *APOB* | Apolipoprotein B-100 |
| *APOC3* | Apolipoprotein C-III |
| *ATPAF2* | ATP synthase mitochondrial F1 complex assembly factor 2 |
| *BAG3* | BAG family molecular chaperone regulator 3 |
| *BMPR1B* | Bone morphogenetic protein receptor type-1B |
| *BMPR2* | Bone morphogenetic protein receptor type II |
| *BRAF* | Serine/threonine-protein kinase B-raf |
| *BSCL2* | Seipin |
| *CACNA1C* | Voltage-dependent L-type calcium channel subunit alpha-1C |
| *CACNA1D* | Voltage-dependent L-type calcium channel subunit alpha-1D |
| *CACNA2D1* | Voltage-dependent calcium channel subunit alpha-2/delta-1 |
| *CACNB2* | Voltage-dependent L-type calcium channel subunit beta-2 |
| *CALM1* | Calmodulin |
| *CALM2* | Calmodulin |
| *CALR3* | Calreticulin 3 |
| *CAPN3* | Calpain-3 |
| *CASQ2* | Calsequestrin-2 |
| *CAV1* | Caveolin-1 |
| *CAV3* | Caveolin-3 |
| *CBL* | E3 ubiquitin-protein ligase CBL |
| *CBS* | Cystathionine beta-synthase |
| *CETP* | Cholesteryl ester transfer protein |
| *COL1A1* | Collagen alpha-1(I) chain |
| *COL1A2* | Collagen alpha-2(I) chain |
| *COL3A1* | Collagen alpha-1(III) chain |
| *COL5A1* | Collagen alpha-1(V) chain |
| *COL5A2* | Collagen alpha-2(V) chain |
| *COQ2* | 4-hydroxybenzoate polyprenyltransferase, mitochondrial |
| *COX15* | Cytochrome c oxidase assembly protein COX15 homolog |
| *COX6B1* | Cytochrome c oxidase subunit 6B1 |
| *CRELD1* | Cysteine-rich with EGF-like domain protein 1 |
| *CRYAB* | Alpha-crystallin B chain |
| *CSRP3* | Cysteine and glycine-rich protein 3 |
| *CTF1* | Cardiotrophin 1 |
| *CTNNA3* | Catenin alpha-3 |
| *DES* | Desmin |
| *DLD* | Dihydrolipoyl dehydrogenase, mitochondrial |
| *DMD* | Dystrophin |
| *DNAJC19* | Mitochondrial import inner membrane translocase subunit TIM14 |
| *DOLK* | Dolichol kinase |
| *DSC2* | Desmocollin 2 |
| *DSG2* | Desmoglein 2 |
| *DSP* | Desmoplakin |
| *DTNA* | Dystrobrevin alpha |
| *ELN* | Elastin |
| *EMD* | Emerin |
| *ENG* | Endoglin |
| *EYA4* | Eyes absent homolog 4 |
| *FAH* | Fumarylacetoacetase |
| *FBN1* | Fibrillin 1 |
| *FBN2* | Fibrillin 2 |
| *FHL1* | Four and a half LIM domains protein 1 |
| *FHL2* | Four and a half LIM domains 2 |
| *FHOD3* | FH1/FH2 domain-containing protein 3 |
| *FKRP* | Fukutin-related protein |
| *FKTN* | Fukutin |
| *FLNA* | Filamin-A |
| *FLNC* | Filamin-C |
| *FOXD4* | Forkhead box protein D4 |
| *GAA* | Lysosomal alpha-glucosidase |
| *GATA4* | Transcription factor GATA-4 |
| *GATA6* | Transcription factor GATA-6 |
| *GATAD1* | GATA zinc finger domain-containing protein 1 |
| *GDF2* | Growth/differentiation factor 2 |
| *GFM1* | Elongation factor G, mitochondrial |
| *GJA1* | Gap junction alpha-1 protein |
| *GJA5* | Gap junction alpha-5 protein |
| *GLA* | Alpha-galactosidase A |
| *GLB1* | Beta-galactosidase |
| *GNPTAB* | N-acetylglucosamine-1-phosphotransferase subunits alpha/beta |
| *GPD1L* | Glycerol-3-phospate dehydrogenase 1-like protein |
| *GUSB* | Beta-glucuronidase |
| *HCN4* | Potassium/sodium hyperpolarization-activated cyclic nucleotide-gated channel 4 |
| *HFE* | Hereditary hemochromatosis protein |
| *HRAS* | GTPase HRas |
| *JAG1* | Jagged-1 |
| *JPH2* | Junctophilin 2 |
| *JUP* | Junction plakoglobin |
| *KCNA5* | Potassium voltage-gated channel subfamily A member 5 |
| *KCND3* | Potassium voltage-gated channel subfamily D member 3 |
| *KCNE1* | Potassium voltage-gated channel subfamily E member 1 |
| *KCNE1L* | Potassium voltage-gated channel subfamily E member 1-like protein |
| *KCNE2* | Potassium voltage-gated channel subfamily E member 2 |
| *KCNE3* | Potassium voltage-gated channel subfamily E member 3 |
| *KCNH2* | Potassium voltage-gated channel subfamily H member 2 |
| *KCNJ2* | Inward rectifier potassium channel 2 |
| *KCNJ5* | G protein-activated inward rectifier potassium channel 4 |
| *KCNJ8* | ATP-sensitive inward rectifier potassium channel 8 |
| *KCNK3* | Potassium channel subfamily K member 3 |
| *KCNQ1* | Potassium voltage-gated channel subfamily KQT member 1 |
| *KLF10* | Krueppel-like factor 10 |
| *KRAS* | GTPase KRas |
| *LAMA2* | Laminin subunit alpha-2 |
| *LAMA4* | Laminin subunit alpha-4 |
| *LAMP2* | Lysosome-associated membrane glycoprotein 2 |
| *LDB3* | LIM domain-binding protein 3 |
| *LDLR* | Low density lipoprotein receptor |
| *LIAS* | Lipoyl synthase, mitochondrial |
| *LMNA* | Prelamin-A/C |
| *LRP6* | Low-density lipoprotein receptor-related protein 6 |
| *MAP2K1* | Dual specificity mitogen-activated protein kinase kinase 1 |
| *MAP2K2* | Dual specificity mitogen-activated protein kinase kinase 2 |
| *MIB1* | E3 ubiquitin-protein ligase MIB1 |
| *MLYCD* | Malonyl-CoA decarboxylase, mitochondrial |
| *MRPL3* | 39S ribosomal protein L3, mitochondrial |
| *MRPS22* | 28S ribosomal protein S22, mitochondrial |
| *MTO1* | Protein MTO1 homolog, mitochondrial |
| *MURC* | Muscle-related coiled-coil protein |
| *MYBPC3* | Myosin-binding protein C, cardiac-type |
| *MYH11* | Myosin, heavy chain 11, smooth muscle |
| *MYH6* | Myosin, heavy chain 6, cardiac muscle, alpha |
| *MYH7* | Myosin, heavy chain 7, cardiac muscle, beta |
| *MYL2* | Myosin regulatory light chain 2, ventricular/cardiac muscle isoform |
| *MYL3* | Myosin light chain 3 |
| *MYLK* | Myosin light chain kinase, smooth muscle |
| *MYLK2* | Myosin light chain kinase 2, skeletal/cardiac muscle |
| *MYOT* | Myotilin |
| *MYOZ2* | Myozenin 2 |
| *MYPN* | Myopalladin |
| *NEBL* | Nebulette |
| *NEXN* | Nexilin |
| *NKX2-5* | Homeobox protein Nkx-2.5 |
| *NOTCH1* | Neurogenic locus notch homolog protein 1 |
| *NOTCH3* | Neurogenic locus notch homolog protein 3 |
| *NPPA* | Atrial natriuretic factor |
| *NRAS* | GTPase NRas |
| *OBSL1* | Obscurin-like protein 1 |
| *PCSK9* | Proprotein convertase subtilisin/kexin type 9 |
| *PDHA1* | Pyruvate dehydrogenase E1 component subunit alpha, somatic form, mitochondrial |
| *PDLIM3* | PDZ and LIM domain protein 3 |
| *PHKA1* | Phosphorylase b kinase regulatory subunit alpha, skeletal muscle isoform |
| *PITX2* | Pituitary homeobox 2 |
| *PKP2* | Plakophilin 2 |
| *PLN* | Cardiac phospholamban |
| *PLOD1* | Procollagen-lysine,2-oxoglutarate 5-dioxygenase 1 |
| *PMM2* | Phosphomannomutase 2 |
| *PRDM16* | PR domain zinc finger protein 16 |
| *PRKAG2* | 5'-AMP-activated protein kinase subunit gamma-2 |
| *PRKG1* | cGMP-dependent protein kinase 1 |
| *PSEN1* | Presenilin-1 |
| *PSEN2* | Presenilin 2 |
| *PTPN11* | Tyrosine-protein phosphatase non-receptor type 11 |
| *RAF1* | RAF proto-oncogene serine/threonine-protein kinase |
| *RANGRF* | Ran guanine nucleotide release factor |
| *RBM20* | Probable RNA-binding protein 20 |
| *RYR2* | Ryanodine receptor 2 |
| *SCN10A* | Sodium channel protein type 10 subunit alpha |
| *SCN1B* | Sodium channel subunit beta-1 |
| *SCN2B* | Sodium channel subunit beta-2 |
| *SCN3B* | Sodium channel subunit beta-3 |
| *SCN4B* | Sodium channel subunit beta-4 |
| *SCN5A* | Sodium channel protein type 5 subunit alpha |
| *SGCA* | Alpha-sarcoglycan |
| *SGCB* | Beta-sarcoglycan |
| *SGCD* | Delta-sarcoglycan |
| *SHOC2* | Leucine-rich repeat protein SHOC-2 |
| *SKI* | Ski oncogene |
| *SLC22A5* | Solute carrier family 22 member 5 |
| *SLC25A4* | ADP/ATP translocase 1 |
| *SLC2A10* | Solute carrier family 2, facilitated glucose transporter member 10 |
| *SLMAP* | Sarcolemmal membrane-associated protein |
| *SMAD1* | Mothers against decapentaplegic homolog 1 |
| *SMAD3* | Mothers against decapentaplegic homolog 3 |
| *SMAD4* | Mothers against decapentaplegic homolog 4 |
| *SMAD9* | Mothers against decapentaplegic homolog 9 |
| *SNTA1* | Alpha-1-syntrophin |
| *SOS1* | Son of sevenless homolog 1 |
| *SPRED1* | Sprouty-related, EVH1 domain-containing protein 1 |
| *SURF1* | Surfeit locus protein 1 |
| *TAZ* | Tafazzin |
| *TBX1* | T-box transcription factor TBX1 |
| *TBX20* | T-box transcription factor TBX20 |
| *TBX5* | T-box transcription factor TBX5 |
| *TCAP* | Telethonin |
| *TGFB2* | Transforming growth factor beta-2 |
| *TGFB3* | Transforming growth factor, beta 3 |
| *TGFBR1* | TGF-beta receptor type-1 |
| *FBR2* | TGF-beta receptor type-2 |
| *TMEM43* | Transmembrane protein 43 |
| *TMEM70* | Transmembrane protein 70, mitochondrial |
| *TMPO* | Thymopoietin |
| *TNNC1* | Troponin C, slow skeletal and cardiac muscles |
| *TNNI3* | Troponin I, cardiac muscle |
| *TNNT2* | Troponin T, cardiac muscle |
| *TPM1* | Tropomyosin alpha-1 chain |
| *TRDN* | Triadin |
| *TRIM63* | E3 ubiquitin-protein ligase TRIM63 |
| *TRPM4* | Transient receptor potential cation channel subfamily M member 4 |
| *TSFM* | Elongation factor Ts, mitochondria |
| *TTN* | Titin |
| *TTR* | Transthyretin |
| *TXNRD2* | Thioredoxin reductase 2, mitochondrial |
| *VCL* | Vinculin |

**Table S1: Enrichment of TBX20tv in different subgroups**

|  | **DCM (isolated and with hypertrabeculation)** | **Isolated LVNC** | **DCM/LVNC** | **Isolated DCM** | **DCM with hypertrabeculation** |
| --- | --- | --- | --- | --- | --- |
| **Probands with phenotype** | 6,536 | 927 | 7,463 | 6308 | 228 |
| **Probands with TBX20tv** | 17 | 7 | 24 | 5 | 12 |
| **% of phenotype** | 0.26% | 0.75% | 0.32% | 0.08% | 5.26% |
| **OR TBX20tv vs internal controls** | 59.38  (95% CI:7.90-446.34);  p = 0.0001 | 173.27  (95% CI:21.29-1409.84);  p < 0.0001 | 73.23  (95% CI: 9.90-541.45);  p < 0.0001 | 18.06  (95% CI:2.11-154.68);  p = 0.0082 | 1265.17  (95% CI:163.78-9773.08);  p < 0.0001 |
| **OR TBX20tv vs gnomAD** | 80.69  (95% CI:27.14-239.88);  p < 0.0001 | 236.06  (95% CI:68.98-807.77);  p < 0.0001 | 99.76  (95% CI: 34.60-287.72);  p < 0.0001 | 24.61 (95% CI:6.60-91.69);  p < 0.0001 | 1723.58  (95% CI:551.53-5386.29);  p < 0.0001 |

**Table S2: TBX20 variants detected in the study**

| **Proteic (NP_001071121.1)** | **cDNA**  **(NM_001077653.2)** | **Chromosomic**  **(NC_000007.13)** | **Exon/Intron Location** | **Variant Type** | **GnomAD freq.**  **(%)** | **HIC**  **freq.**  **(%)** | **Index**  **cases** | **Carriers** | **ACMG**  **rules applied** | **Final ACMG Patog.** |
| --- | --- | --- | --- | --- | --- | --- | --- | --- | --- | --- |
| p.His225Glnfs*22 | c.675delT | g.35280629delA | Exon 5 | Frameshift | 0.00 | 0.007 | 2 | 18 | PVS1, PP1_Strong, PM2, PS4_Supporting, | P |
| p.Gln407* | c.1219C>T | g.35242167G>A | Exon 8 | Nonsense | 0.00 | 0.003 | 1 | 1 | PVS1, PM2, | LP |
| p.Pro399fs | c.1196_1197delCA | g.35242189_35242190delTG | Exon 8 | Frameshift | 0.00 | 0.003 | 1 | 2 | PVS1, PM2, | LP |
| p.Trp169* | c.507G>A | g.35288327C>T | Exon 3 | Nonsense | 0.00 | 0.003 | 1 | 2 | PVS1, PM2, | LP |
| p.Thr192Tyrfs*3 | c.573_574insT | g.35284645_35284646insA | Exon 4 | Frameshift | 0.00 | 0.003 | 1 | 5 | PVS1,PM2,PP1, | P |
| Del exon 7_8 | c.(890+1_891-1)_(*1_?)del | g.(?_35242041)_(35244195_35271115)del | Exon 7_8 | CNV | 0.00 | 0.003 | 1 | 3 | PVS1, PM2, | LP |
| p.Thr89Serfs*30 | c.264_270delCACCCCC | g.35289674_35289680delGGGGTGG | Exon 2 | Frameshift | 0.00 | 0.003 | 1 | 1 | PVS1, PM2, | LP |
|  | c.655-2A>G | g.35280651T>C | Intron 4 | Splicing | 0.00 | 0.007 | 2 | 4 | PVS1, PM2, PP1, PS4_Supporting, | P |
| Ile91Serfs*30 | c.270delC | g.35289677delG | Exon 2 | Frameshift | 0.00 | 0.003 | 1 | 1 | PVS1, PM2, | LP |
|  | c.380+1G>A | g.35289562C>T | Intron 2 | Splicing | 0.00 | 0.003 | 1 | 1 | PVS1, PM2, | LP |
| p.*448Cys | c.1344A>C | g.35242042T>G | Exon 8 | Stop loss | 0.00 | 0.003 | 1 | 3 | PVS1, PM2, | LP |
| p.Arg334* | c.1000C>T | g.35244085G>A | Exon 7 | Nonsense | 0.00 | 0.003 | 1 | 4 | PVS1,PM2,PP1, | P |
|  | c.545+1G>A | g.35288288C>T | Intron 3 | Splicing | 0.00 | 0.003 | 1 | 1 | PVS1, PM2, | LP |
|  | c.381-2A>C | g.35288455T>G | Intron 2 | Splicing | 0.00 | 0.003 | 1 | 3 | PVS1, PM2, | LP |
|  | c.381-1G>A | g.35288454C>T | Intron 2 | Splicing | 0.00 | 0.003 | 1 | 1 | PVS1, PM2, | LP |
| p.Lys198Thrfs*6 | c.593_594delAA | g.35284622_35284623delTT | Exon 4 | Frameshift | 0.00 | 0.003 | 1 | 1 | PVS1, PM2, | LP |
| p.Thr89Hisfs*7 | c.264_265insC | g.35289680_35289681insG | Exon 2 | Frameshift | 0.00 | 0.003 | 1 | 3 | PVS1, PM2, | LP |
| p.Thr370Ilefs*18 | c.1109_1119delCCAGCACAGCA | g.35242270_35242280delTGTGCTGGTGC | Exon 8 | Frameshift | 0.00 | 0.003 | 1 | 2 | PVS1, PM2, | LP |
| p.Ser302Thrfs*2 | c.905delG | g.35244180delC | Exon 7 | Frameshift | 0.00 | 0.003 | 1 | 1 | PVS1, PM2, | LP |

**Table S3: Other potentially relevant variants identified in the present study**

| **Gene** | **Proteic** | **cDNA** | **Chromosomic** | **Exon/Intron Location** | **Variant Type** | **GnomAD freq.**  **(%)** | **HIC**  **freq.**  **(%)** | **Index**  **cases** | | **Carriers** | | **ACMG**  **rules applied** | **Final ACMG Patog.** |
| --- | --- | --- | --- | --- | --- | --- | --- | --- | --- | --- | --- | --- | --- |
| MYBPC3 | p.Arg810His | NM_000256.3:c.2429G>A | chr9:47359115C>T | 25 | Missense | 0.005 | 0.076 | 1 | 2 | | PS4, PM2, PP3, | | LP |
| MYBPC3 |  | NM_000256.3:c.927-9G>A | chr9:47367930C>T | 11 | Splicing | 0.002 | 0.049 | 1 | 3 | | PVS1, PS4, PS3, PM2, PP1, | | P |
| MYH7 | p.Ala1394Thr | NM_000257.3:c.4180G>A | chr14:23886885C>T | 31 | Missense | 0.001 | 0.008 | 1 | 3 | | PM2, PP3 | | VUS |
| KCNQ1 | p.Ala300Thr | NM_000218.2:c.898G>A | chr11:.2594193G>A | 6 | Missense | 0.005 | 0.129 | 1 | 4 | | PS4, PM2, PS3 | | P* |

* Pathogenic with recessive inheritance pattern (in homozygosis or compound heterozygosis

**Table S4: Clinical characteristics of all the carriers**

| **FAMILY** | **SUBJECT** | **SEX** | **GENETIC VARIANT** | **DIAGNOSIS/FU AGE** | **PHENOTYPE** | **Initial LVEF (%)** | **Initial LVEDD (mm)** | **Final LVEF (%)** | **Final LVEDD (mm)** | **SYMPTOMS** | **ECG** | **EVENTS** | **CHARACTERISTICS** |
| --- | --- | --- | --- | --- | --- | --- | --- | --- | --- | --- | --- | --- | --- |
| 133461 | IV.1 | Male | p.His225Glnfs*22 | 9/35 | LVNC + CHD | 68 | 49 | 64 | 46 | Progressive dyspnea. Cardiogenic shock | AF | ICD-CRT (29y). Heart transplant (35 y) | LVNC with restriction. BAV. LGE (+). |
|  | III.4 | Female | p.His225Glnfs*22 | 57/63 | DCM | 25 | 54 | 40 | 42 | NYHA II | SR | CRT (59y) |  |
|  | IV.2 | Female | p.His225Glnfs*22 | 34/38 | LVNC | 74 | 46 | 72 | 45 | NYHA II | SR |  | LGE (+) |
|  | V.1 | Male | p.His225Glnfs*22 | 11/11 | Unaffected |  |  |  |  | Asymptomatic |  |  |  |
|  | II.1 | Male | p.His225Glnfs*22 |  | DCM+LVNC |  |  |  |  |  |  |  | LVNC+DCM with severe dysfunction. Revascularized (ischemic heart disease). |
| 33598 | IV.9 | Male | p.His225Glnfs*22 | 4/12 | LVNC + CHD | 59 | 41 | 65 | 53 | Asymptomatic | SR |  | Mild mitral valve prolapse. Isolated ESV and EV. Z-score last echo: +1.05. |
|  | III.13 | Female | p.His225Glnfs*22 | 38/44 | LVNC | 68 | 41 |  |  | Asymptomatic | SR |  | Normal echocardiogram, MRI compatible with non-compaction. Isolated EV without clinical repercussions. |
|  | III.6 | Female | p.His225Glnfs*22 | 45/45 |  |  |  |  |  |  |  |  |  |
|  | III.9 | Female | p.His225Glnfs*22 | 40/45 | Unaffected | 60 |  | 60 |  | Asymptomatic | SR |  |  |
|  | III.10 | Male | p.His225Glnfs*22 | 30/37 | LVNC | 60 | 54 | 56 | 55 | Palpitations | SR |  | MRI compatible with non-compaction. Right posteroseptal accessory pathway, ablation (x3) for symptomatic supraventricular tachycardia. |
|  | IV.8 | Male | p.His225Glnfs*22 | 1/1 | LVNC | 60 |  |  |  | Asymptomatic | SR |  | Normal Echocardiogram and Holter-ECG |
|  | IV.3 | Male | p.His225Glnfs*22 | 12/18 | Unaffected | 62 |  |  |  |  |  |  | Normal Echocardiogram and MRI |
|  | II.5 | Female | p.His225Glnfs*22 | 53/75 | LVNC | 57 | 50 | 62 | 54 | Asymptomatic | SR |  | MRI compatible with non-compaction. No LGE |
|  | II.1 | Male | p.His225Glnfs*22 | 59/74 | DCM+LVNC | 45 |  | 15 | 74 | Progressive dyspnea. Refractory CHF | AF | ICD-CRT (74y). Cardiovascular death (74y) | Non-ischemic DCM with severe dilatation and dysfunction. Apical thrombus. Extensive septal scar on the posterobasal aspect. Moderate-severe pulmonary hypertension. |
|  | III.2 | Male | p.His225Glnfs*22 | 46/51 | LVNC | 55 | 47 | 55 | 48 | Asymptomatic | SR |  | LA and RV slightly dilated. Mitral valve with mild restriction. |
|  | III.3 | Female | p.His225Glnfs*22 | 44/49 | Unaffected | 55 |  | 55 |  | Asymptomatic | SR |  | LBBB. Self-limited episodes of wide-QRS tachycardia |
|  | IV.1 | Male | p.His225Glnfs*22 | 12/17 | LVNC | 58 | 42 | 65 | 42 | Dyspnea NYHA II | SR |  | No compaction in LV free wall and apex. RV free wall hypertrabeculation with RVEF 48%, short PR on Holter. |
|  | II.8 | Female | p.His225Glnfs*22 |  |  |  |  |  |  |  |  |  |  |
| 36796 |  | Male | p.Gln407* | 57/74 | DCM+LVNC | 43 | 58 | 26 | 61 | Dyspnea | SR | SCD (57y), ICD (secondary prevention) | Progressive LV dilatation and dysfunction. No LGE |
| 30125 | I.1 | Male | p.Pro399fs | 10/48 | DCM + CHD | 55 | 43 | 33 | 41 | Progressive dyspnea. Refractory CHF | AF | AF with peripheral embolism (25y). Cardiac transplantation and cardiovascular death (48y) | Atrial septal defect. Thickened mitral valve with prolapse. Symptomatic CHF since the age of 22, with data of restriction and pulmonary hypertension, atrial fibrillation with peripheral embolism. |
|  | II.1 | Female | p.Pro399fs | 1/25 | DCM+LVNC + CHD | 70 |  | 40 | 35 | Neonatal heart failure | SR |  | VSD. Double outlet RV. Subaortic stenosis. Single coronary artery (surgical correction) |
| 58490 | I.2 | Female | p.Trp169* | 53/57 | LVNC | 53 |  | 50 |  | Chest discomfort | SR |  | LGE (-) |
|  | II.1 | Male | p.Trp169* | 28/33 | LVNC | 60 | 45 | 60 | 45 | Asymptomatic | SR |  | No LGE |
| 101265 | III.1 | Female | p.Thr192Tyrfs*3 | 46/52 | DCM+LVNC + CHD | 50 | 57 | 46 | 52 | Asymptomatic | SR |  | VSD. LGE |
|  | III.3 | Female | p.Thr192Tyrfs*3 | 56/60 | Unaffected | 72 |  | 72 |  | Asymptomatic | SR |  |  |
|  | IV.1 | Male | p.Thr192Tyrfs*3 | 20/22 | LVNC + CDH | 70 | 57 | 60 | 57 | Asymptomatic | SR |  | VSD. No LGE. PR interval at lower limit |
|  | IV.2 | Female | p.Thr192Tyrfs*3 | 16/18 | Unaffected | 70 |  | 70 |  | Asymptomatic | SR |  | Short PR with pre-excitation |
|  | IV.3 | Male | p.Thr192Tyrfs*3 | 25/26 | DCM+LVNC + CHD |  |  |  |  | Asymptomatic | Low atrial rhythm |  | Aortic coarctation. Bicuspid aortic valve. Dilatation and severe ventricular dysfunction |
| 103885 | II.1 | Female | Del exons 7_8 | 32/44 | DCM | 60 | 45 | 46 | 46 | Sudden cardiac death in postpartum | SR | SCD (32Y). ICD (secondary prevention) | Progressive LV dysfunction. No LGE |
|  | II.3 | Female | Del exons 7_8 | 43/47 | LVNC | 50 | 40 | 57 | 38 | Palpitations | SR |  |  |
|  | III.1 | Male | Del exons 7_8 | 20/20 | LVNC | 50 | 45 |  |  | Asymptomatic |  |  |  |
| 84267 |  | Female | p.Thr89Serfs*30 | 54/57 | DCM+LVNC | 40 | 45 | 40 | 45 | Dyspnea NYHA II-III | Paroxysmal FA | ICD-CRT (56Y) | Family history of cardiomyopathy and sudden death in the first degree |
| 53558 |  | Female | c.655-2A>G | 73/78 | DCM+LVNC | 43 | 55 | 44 |  | Dyspnea NYHA II | SR |  | No LGE |
| 35756 | III.1 | Male | c.655-2A>G | 39/48 | LVNC | 60 | 50 | 56 | 49 | Initial NYHA II | SR |  |  |
|  | III.2 | Female | c.655-2A>G | 37/45 | DCM+LVNC | 51 | 50 | 49 | 46 | Asymptomatic | SR |  | No LGE |
|  | II.2 | Male | c.655-2A>G | 65/73 | LVNC | 58 | 44 | 53 | 53 | Asymptomatic | SR |  | Ischemic heart disease and diabetes. LGE |
| 122255 |  | Male | p.Ile91Serfs*30 | 30/31 | DCM+LVNC + CHD | 44 | 54 | 56 | 61 | Cardiac tamponade. Dyspnea | SR |  | ASD. Ablated atrial flutter. No LGE. |
| 64656 |  | Male | c.380+1G>A | 12/16 | LVNC | 65 | 40 |  |  | Asymptomatic | SR |  |  |
| 127563 | II.1 | Female | p*448Cys | 1/20 | DCM+LVNC | 49 | 43 | 55 | 51 | Initial dyspnea II-III | SR |  | No LGE |
|  | I.2 | Female | p*448Cys |  | Unaffected | 69 | 44 |  |  | Asymptomatic | SR |  |  |
|  | II.2 | Female | p*448Cys |  | Unaffected | 63 | 33 | 60 | 45 | Asymptomatic | SR |  |  |
| 116333 | II.1 | Male | p.Arg334* | 45/59 | DCM+LVNC + CHD | 45 | 61 | 46 | 65 | Dyspnea NYHA II | SR |  | Myxomatous mitral valve prolapse A2. No LGE |
|  | II.3 | Female | p.Arg334* | 41/51 | LVNC + CHD | 65 | 52 | 60 | 49 | Dyspnea NYHA II | SR | Chronotropic failure due to sinus dysfunction. Pacemaker implantation | Billowing mitral. No LGE |
|  | II.8 | Female | p.Arg334* | 62 | DCM + CHD | 70 | 53 | 59 | 50 | Asymptomatic | SR |  | Bicuspid aortic valve. Aortic regurgitation II/IV |
|  | III.2 | Female | p.Arg334* | 22 | Unaffected | 73 | 44 | 74 | 46 | Asymptomatic | SR |  |  |
| 107653 |  | Female | c.545+1G>A | 46/53 | DCM+LVNC | 45 | 39 | 52 | 38 | Mild dyspnea. Palpitations | SR | Sinus bradycardia |  |
| 89577 | III.1 | Male | c.381-2A>C | 25/27 | DCM+LVNC + CHD | 25 | 52 | 31 | 58 | Dyspnea NYHA II-III | AF |  | LVNC with restriction. Separate origin of DA and Cx |
|  | II.2 | Female | c.381-2A>C | 50/60 | CHD | 60 |  | 60 | 50 | Asymptomatic | SR |  | Bicuspid aortic valve. Coarctation of the aorta and dilatation of ascending aorta |
|  | III.2 | Female | c.381-2A>C | 26/27 | CHD | 58 |  |  |  | Asymptomatic | SR |  | Bicuspid aortic valve |
| 76525 |  | Male | c.381-1G>A | 71 | DCM | 44 | 64 | 35 | 55 | Dizziness | Biventricular stimulation | CRT (71yo) | Incessant atrial tachycardia, ablation. LGE |
| 88245 | IV.2 | Female | p.Lys198Thrfs*6 | 10/28 | HCM + DCM + CHD | 30 |  | 38 | 53 | Asymptomatic. Screening | SR. NSVT | ICD. Cardiac transplantation (26yo) | Atrial septal defect. Carrier of pathogenic variant in MYBPC3 (inherited from her father). TBX20 variant was found to be de novo |
| 136919 | I.1 | Male | p.Thr89Hisfs*7 | 64/74 | DCM+LVNC + CHD | 50 | 49 | 34 | 63 | Dyspnea NYHA II-III | SR |  | Prolapse both leaflets mitral valve. Moderate-severe MR. LGE |
|  | II.1 | Male | p.Thr89Hisfs*7 | 45 | Unaffected | 60 | 51 | 63 | 50 | Asymptomatic | SR |  | Apical trabeculation without non compaction criteria |
|  | II.2 | Female | p.Thr89Hisfs*7 | 49 | Unaffected | 60 | 42 |  |  | Asymptomatic | SR |  | Apical trabeculation without non compaction criteria |
| 138468 | I.1 | Male | p.Thr370Ilefs*18 | 71/72 | DCM+LVNC | 10 | 57 | 15 | 56 | Asymptomatic | SR |  |  |
|  | II.2 | Male | p.Thr370Ilefs*18 | 49 | Unaffected | 60 | 47 | 50 | 46 | Asymptomatic | SR |  |  |
| 144716 |  | Male | p.Ser302Thrfs*2 | 73/77 | DCM | 15 | 71 | 42 | 61 | Dyspnea NYHA II | SR |  |  |

**Figure S1: Pedigrees of tvTBX20 carriers**


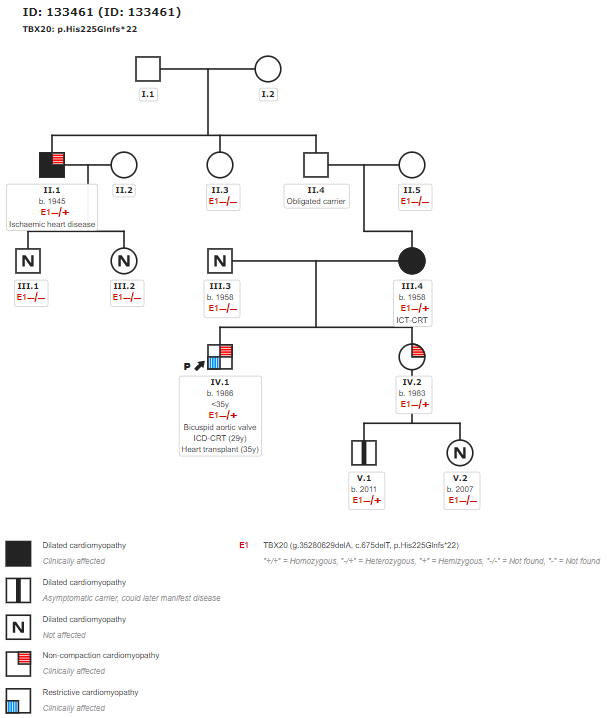

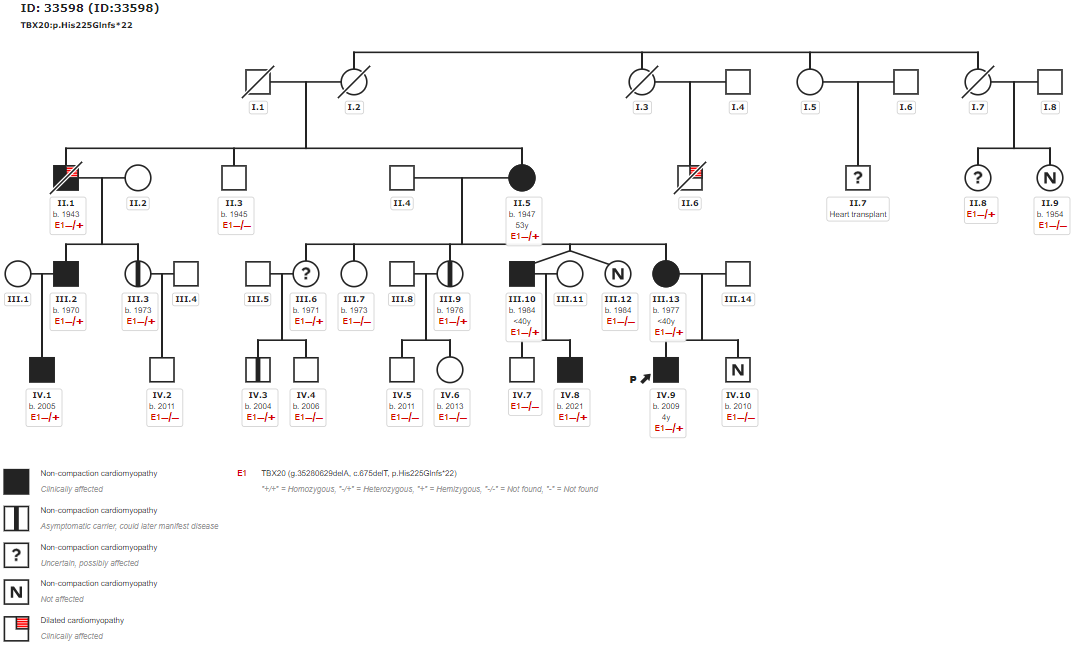


**LOD SCORE=1.68**

**LOD SCORE=0.43**


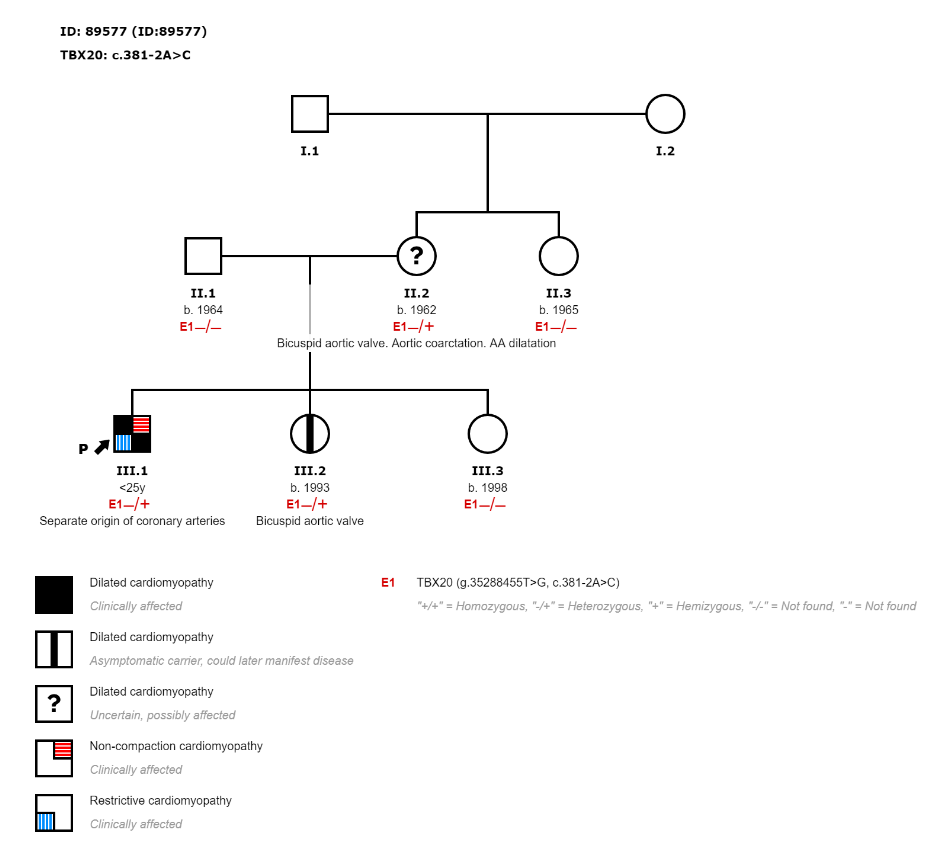

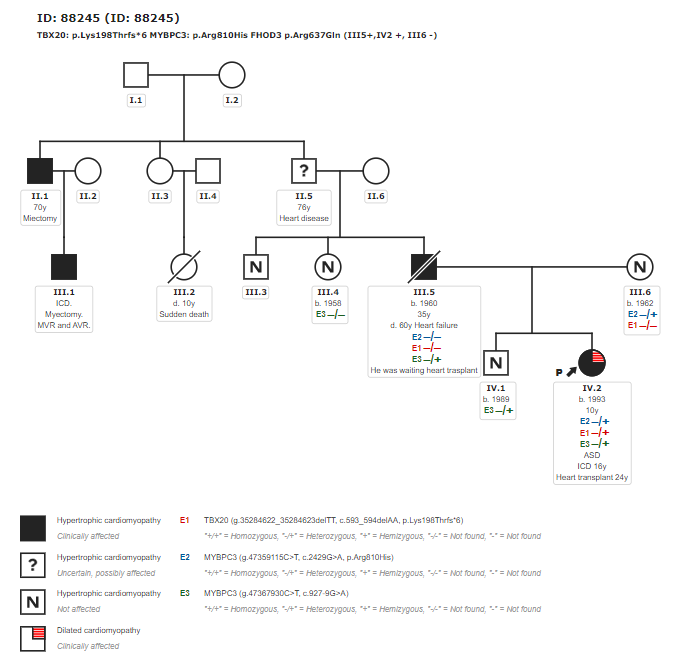


**LOD SCORE=0.30**

**LOD SCORE=N/A**


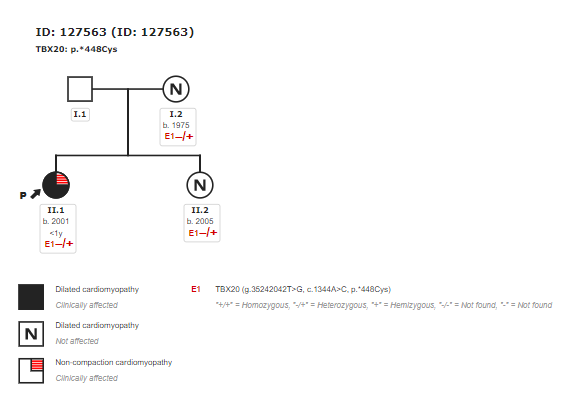

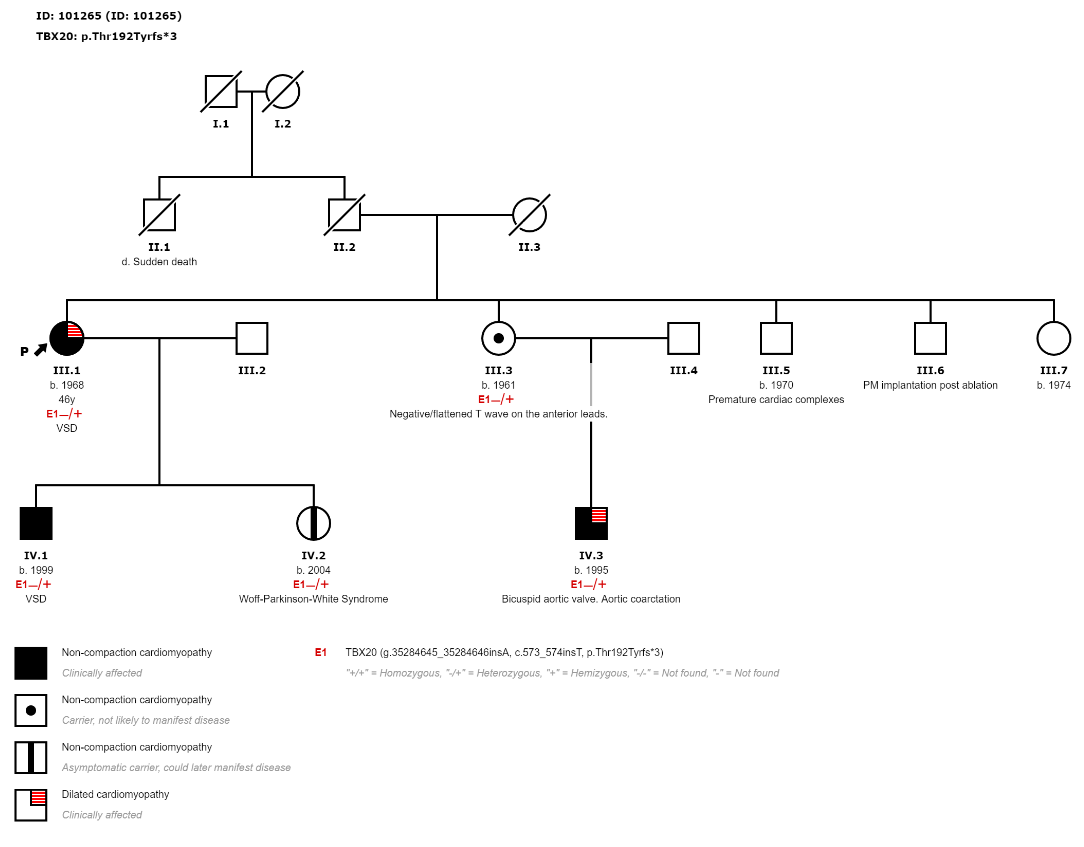


**LOD SCORE=0.00**

**LOD SCORE=0.25**


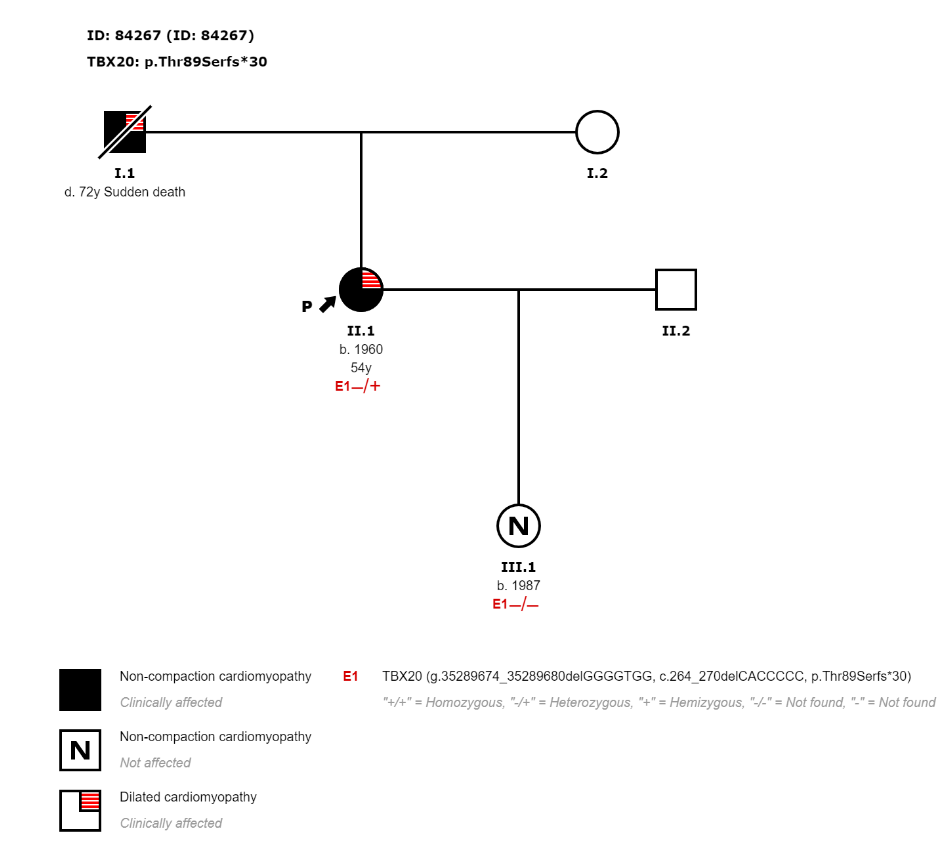

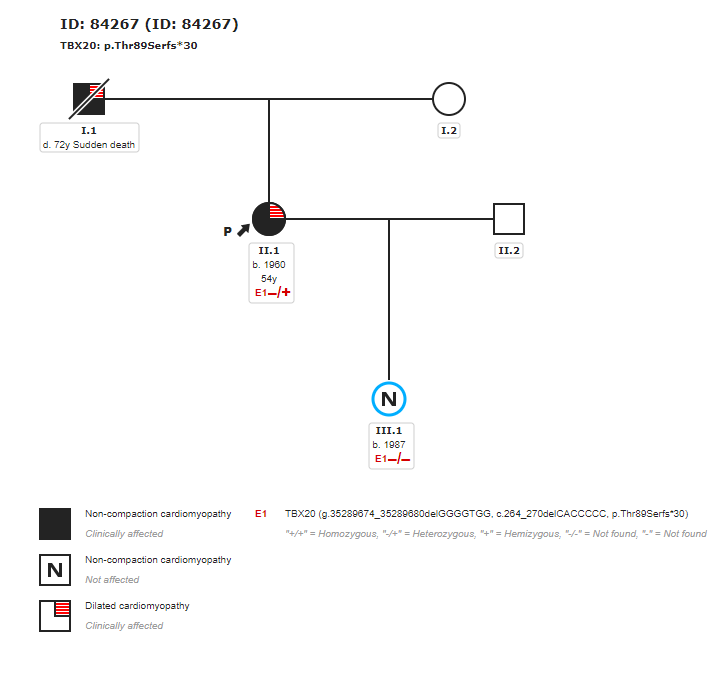


**LOD SCORE=0.08**

**LOD SCORE=0.12**


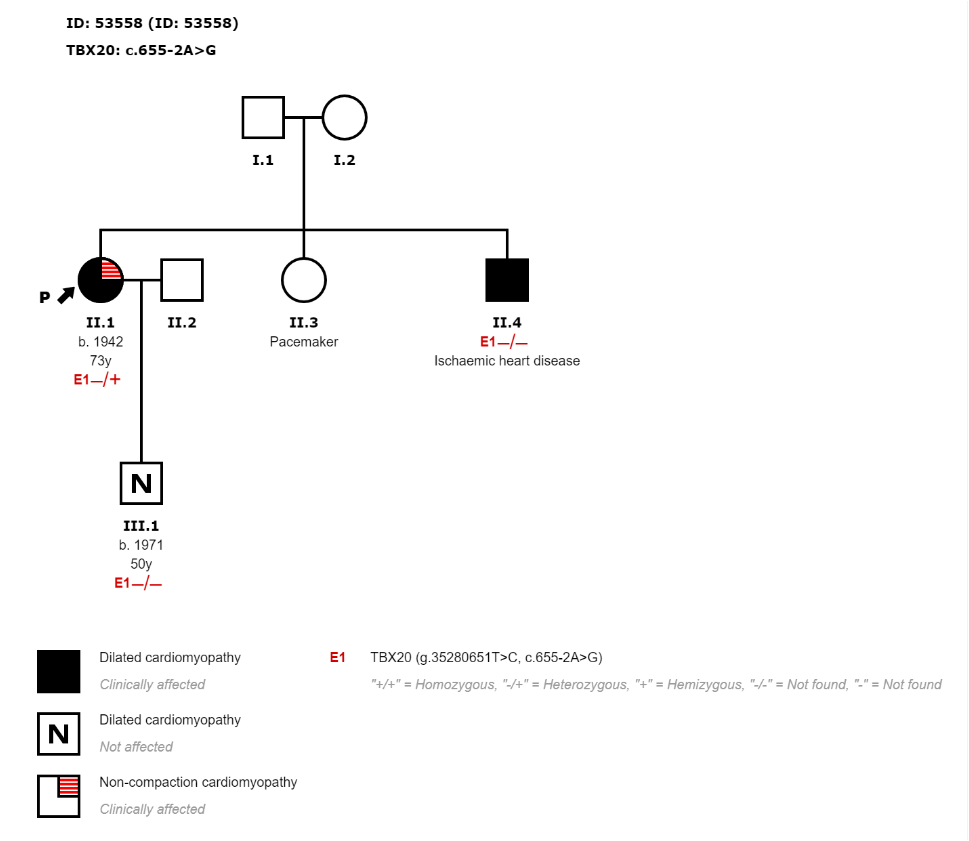

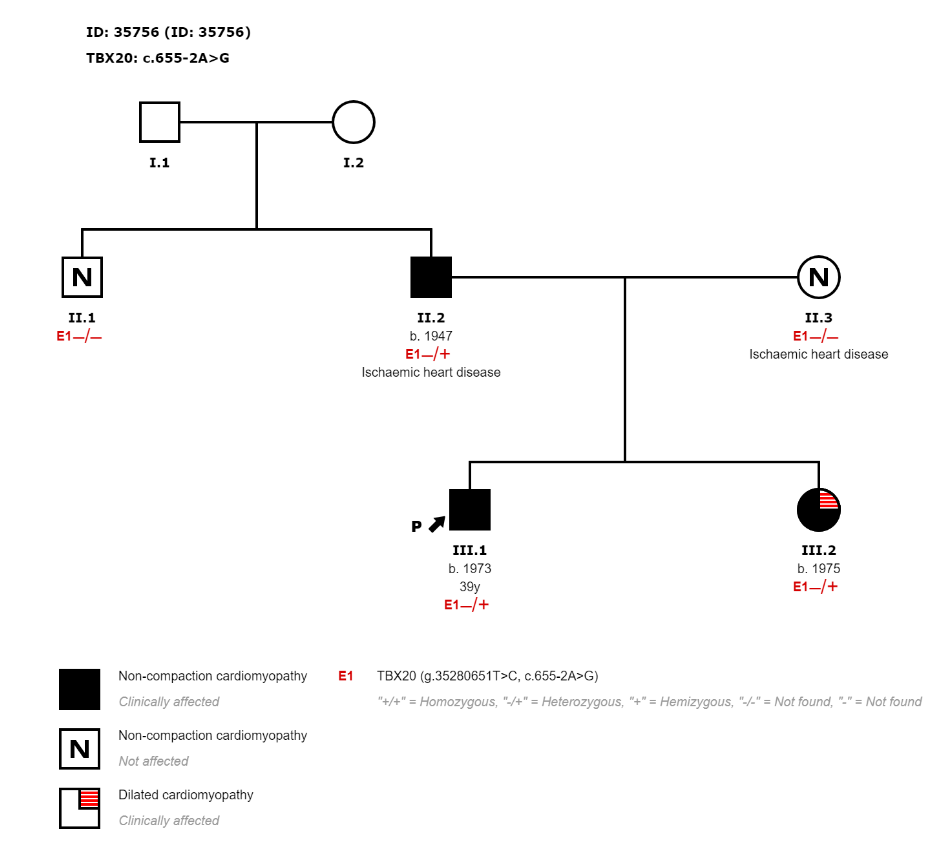


**LOD SCORE=0.60**

**LOD SCORE=0.00**


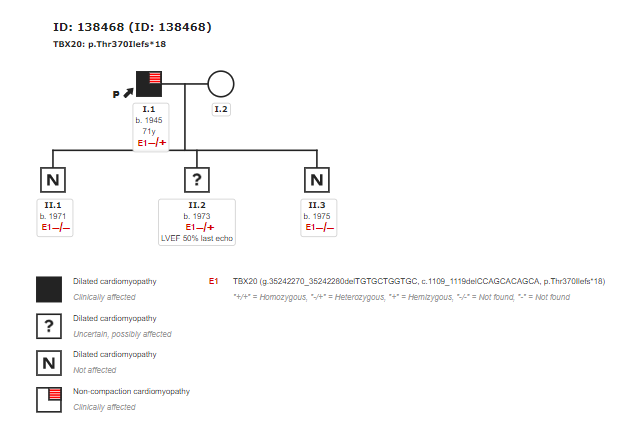

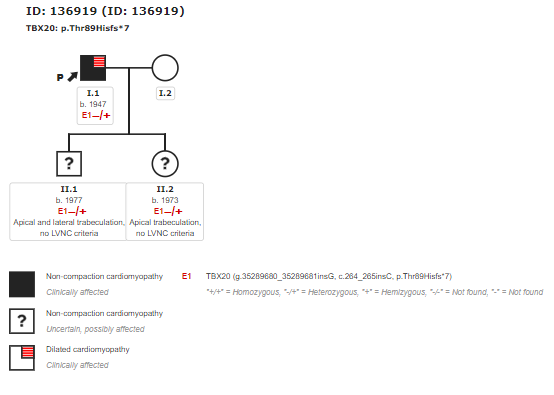


**LOD SCORE=0.00**

**LOD SCORE=0.52**


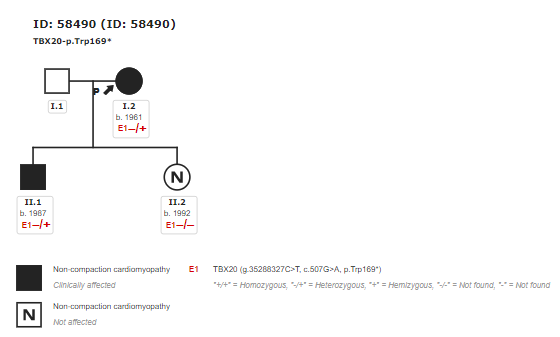

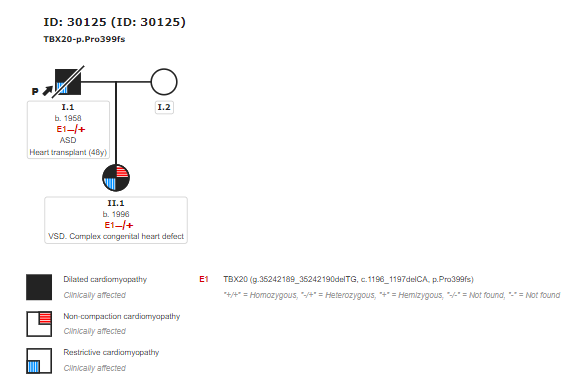


**LOD SCORE=0.17**

**LOD SCORE=0.00**


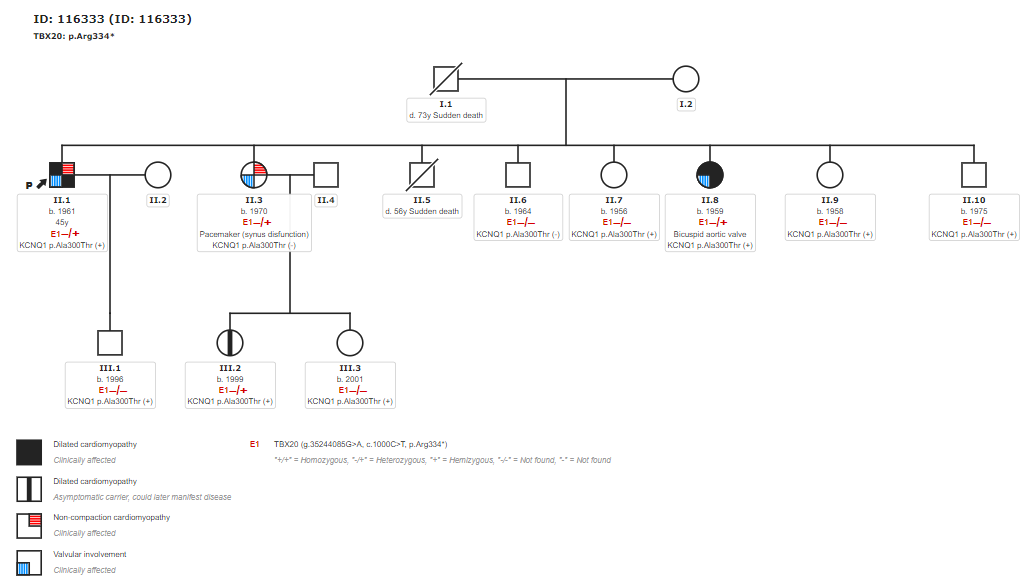


**LOD SCORE=0.38**

**Figure S2: Penetrance according to index status, phenotype, and subtype of cardiomyopathy**


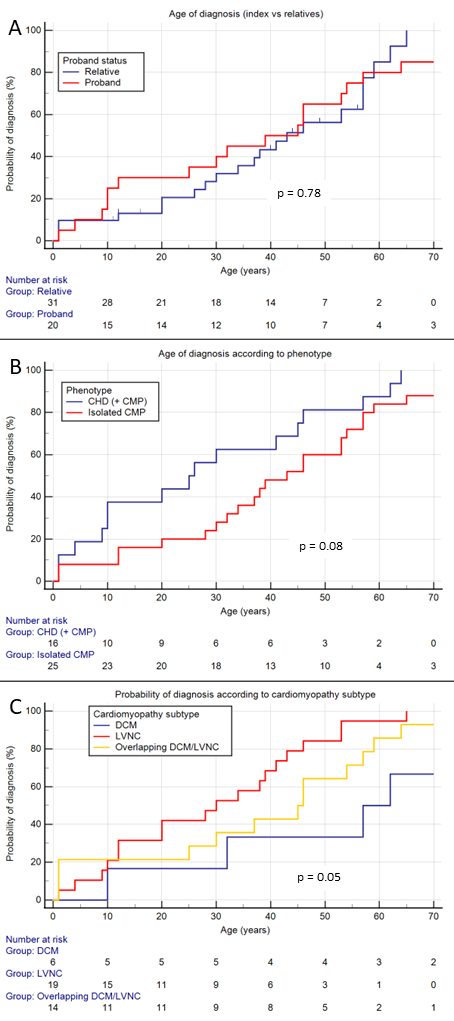


CHD = congenital heart defect; CMP = cardiomyopathy; DCM = dilated cardiomyopathy; LVNC = left ventricular non compaction

**Figure S3: Survival free of MACE**


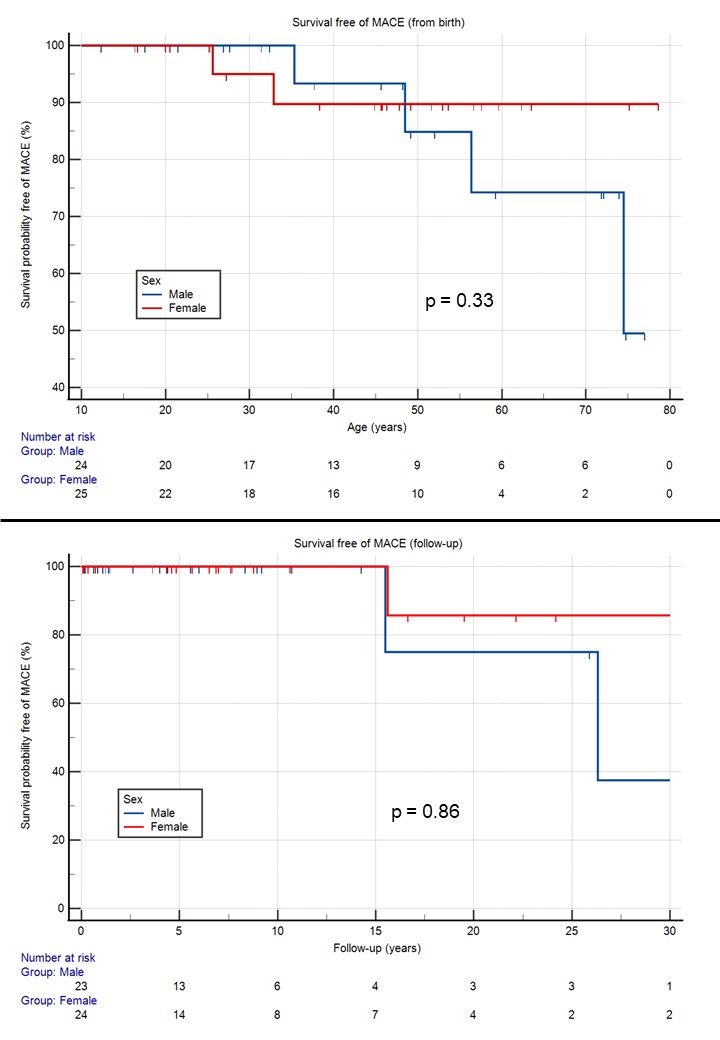

Supplement: Supplementary file 1 [file hcg-17-e004404-s001.docx]
